# Nature-Based Interventions for Autistic Children: A Systematic Review and Meta-Analysis

**DOI:** 10.1001/jamanetworkopen.2023.46715

**Published:** 2023-12-07

**Authors:** Myrian Sze Nga Fan, William Ho Cheung Li, Laurie Long Kwan Ho, Lophina Phiri, Kai Chow Choi

**Affiliations:** 1 The Chinese University of Hong Kong, Hong Kong, China

## Abstract

**Importance:**

There is a gap in the evidence regarding nature-based interventions (NBIs) for children with autism spectrum disorder (ASD).

**Objective:**

To systematically review and meta-analyze available evidence on the health-related outcomes in NBIs for children with ASD.

**Data Sources:**

The Cumulative Index to Nursing and Allied Health Literature, Cochrane, Embase, Emcare, Education Resources Information Center, Global Health, MEDLINE, PsycInfo, SPORTDiscus, and Web of Science were searched from inception until May 2023. Google Scholar and references from included studies were searched for additional studies.

**Study Selection:**

Included studies were randomized clinical trials (RCTs), controlled studies, and single-group before-and-after studies that reported health-related outcomes.

**Data Extraction and Synthesis:**

This review adhered to the Preferred Reporting Items for Systematic Reviews and Meta-Analysis (PRISMA) reporting guidelines. Random-effects meta-analyses were used to synthesize the data. The findings of studies that were ineligible for meta-analysis were summarized according to the Synthesis Without Meta-analysis (SWIM) reporting guidelines.

**Main Outcomes and Measures:**

The outcomes of interest were health-related outcomes (ie, social functioning, behavioral functioning, emotional functioning, sensory functioning) and the self-reported well-being of children with ASD.

**Results:**

A total of 24 studies with 717 participants (mean age range, 5.3 to 17.8 years; 141 [21.9%] female) were included. A meta-analysis from 13 studies indicated a significant negative moderate association between NBIs and social communication (standardized mean difference [SMD], −0.59; 95% CI, −0.85 to −0.34). For behavioral functioning outcomes, NBIs showed a significant moderate association with reduced hyperactivity (SMD, −0.56; 95% CI, −0.86 to −0.26) and a small to moderate association with reduced irritability (SMD, −0.49; 95% CI, −0.79 to −0.19). For sensory functioning, NBIs were significantly associated with improved inattention and distractibility (SMD, 1.13; 95% CI, 0.67 to 1.60). Significant moderate associations were observed in sensory seeking (SMD, 0.77; 95% CI, 0.33 to 1.22; *P* < .001; *I*^2^ = 0%) and sensory sensitivity (SMD, 0.56; 95% CI, 0.12 to 1.00; *P* = .01; *I*^2^ = 0%). Heterogeneity of the intervention effects was not high, and *I*^2^ ranged from 0% to 67%.

**Conclusions and Relevance:**

The findings of this systematic review and meta-analysis suggested an association of NBIs in group-based recreational therapy with experiential learning with positive short-term outcomes on sensory, social, and behavioral functioning for children with ASD. Future evidence using robust study design to aid the health and functional trajectories of children with ASD is recommended.

## Key Points

**Question:**

Are nature-based interventions (NBIs) associated with health-related functional trajectories of children with autism spectrum disorder (ASD)?

**Findings:**

This systematic review and meta-analysis, including 24 studies with 717 participants, found that NBIs in the form of group-based recreational therapy with experiential learning were associated with short-term improvement in sensory, social, and behavioral functioning among children with ASD.

**Meaning:**

These findings suggest that NBIs, as an alternative approach outside the traditional natural environment such as clinic, home, and education settings, were associated with improvements in health-related functioning outcomes of children with ASD.

## Teaser

This systematic review and meta-analysis assesses the available evidence on the associations of nature-based interventions with health-related functional outcomes among children with autism spectrum disorder.

## Introduction

Autism spectrum disorder (ASD) is a complex neurodevelopmental condition that is characterized by the *Diagnostic and Statistical Manual of Mental Disorders* (Fifth Edition) (*DSM-5*)^1^ as “persistent deficits in social communication and social interaction” with the presence of restricted, repetitive patterns of behavior, interests, or activities. Psychosocial and behavioral interventions for children with ASD are designed to promote learning, participation, and skill development while focusing on building strengths and supporting individual growth^2^; however, data are lacking concerning the primary purpose of all interventions, which is the subjective well-being of the population.^3,4,5^ The 2021 Lancet Commission recommended that identifying effective short-term personalized interventions in randomized clinical trials (RCTs) in settings outside clinics is necessary to improve the lives of individuals with ASD.^6^ Clinics are the most common intervention setting for children with ASD, followed by homes and education settings.^4^

Nonpharmacological interventions can be summarized in a taxonomy comprising 9 categories^2^: behavioral interventions, developmental interventions, naturalistic development behavioral interventions, sensory-based interventions, technology-based interventions, animal-assisted interventions, cognitive behavioral therapy (CBT), Treatment and Education of Autistic and Related Communication-Handicapped Children (TEACCH) programs, and other interventions that do not fit within these categories. This taxonomy has been adopted by updated reviews.^3,4,5,7,8,9^ The reference to the *natural environment* in conventional interventions typically pertains to familiar settings, such as homes and education settings.

Nature-based interventions (NBIs) are professional-led, purposeful approaches that enhance health and well-being by actively involving individuals in structured, nature-based experiences tailored to address specific needs and achieve defined objectives, such as quality of life,^10^ general well-being,^11^ mental well-being,^12^ psychological well-being,^13^ self-efficacy, and life satisfaction.^7,14,15,16,17^ NBIs for children with ASD in the existing literature^18,19,20,21,22,23,24,25,26,27,28,29,30,31,32,33,34,35,36^ are typically classified either as animal-assisted interventions or categorized under the other category.^2^ Evidence of improved health-related outcomes following interacting with nature has been demonstrated in different populations.^14,37,38,39,40,41,42,43^ However, to our knowledge, no systematic review of NBIs has been conducted that explicitly focused on children with ASD. Based on the available literature, NBIs have the potential to address health and functional outcome gaps among children with ASD, particularly regarding subjective well-being.

The World Health Organization (WHO), in its International Classification of Functioning Disability and Health (ICF), highlights the need to address environmental factors and promote participation for individuals with ASD,^4,44^ particularly within the natural environment.^45^ While the physical form of the natural environment may appear similar in conventional interventions, there are contextual differences in NBIs. The natural environment of NBIs refers to outdoor green or blue space.^16^ Depending on the level of engagement with nature, the participation setting of NBIs can be regarded as the natural environment for children with ASD. By summarizing the potential benefits of NBIs for children with ASD and emphasizing direct interaction with nature in the natural environment, there is an opportunity to expand the natural environment to include community settings. As evidence to guide NBIs is currently limited,^44^ this systematic review and meta-analysis aims to summarize existing evidence to support the future development of NBIs, focusing on study designs, outcomes, and delivery, to address the knowledge gaps on environmental factors and the outcome gaps in subjective well-being.

## Methods

The conduct and reporting of this systematic review and meta-analysis adhered to the Preferred Reporting Items for Systematic Reviews and Meta-analyses (PRISMA) reporting guideline. The review protocol was registered in the PROSPERO database (identification No. CRD42023411787).

### Eligibility Criteria

All NBIs that used relevant experimental controlled trials to improve health-related outcomes among children with ASD were considered for inclusion in our review. To be eligible, the health-related outcomes had to be measured for at least 1 of the primary or secondary outcomes reported. NBIs must occur in a natural outdoor setting, known as *outdoor green* or *blue spaces*.^16^ Population, intervention, control, and outcomes data are presented in eTable 1 in Supplement 1.

### Study Selection and Data Extraction

We conducted a comprehensive search in 10 English electronic bibliographic databases, Cumulative Index to Nursing and Allied Health Literature, Cochrane, Embase, Emcare, Education Resources Information Center, Global Health, Medline, PsycInfo, SPORTDiscus, and Web of Science, from inception to May 11, 2023. Search strategies with specific search terms and search results are presented in eTables 2 to 12 in Supplement 1. No limits on the publication year were applied, and only peer-reviewed articles in English were included. Google Scholar (Alphabet) and references from the identified articles were checked manually for additional potential studies. All records retrieved by electronic searching were imported to EndNote version 20 (Clarivate). Duplicates were removed after exporting all records to a review management website (Covidence) for screening and review. Titles and abstracts were screened against the eligibility criteria by 2 independent reviewers (M.S.N.F. and L.P.), followed by full-text screening. A third reviewer (L.L.K.H) was consulted in cases of diverging opinions. Data were independently extracted from eligible studies by 2 reviewers (M.S.N.F. and L.P.) using a shared extraction form. A third reviewer (L.L.K.H) was consulted in cases of disagreement. To evaluate the outcomes of NBIs, raw means and SDs were extracted for the intervention and control groups to calculate standardized mean differences (SMDs). Data were collected and analyzed from each study at the immediate postintervention time point, and, if indicated, at follow-up evaluation time points.

### Study Risk of Bias Assessment

Methodological quality was evaluated independently by 2 reviewers (M.S.N.F. and L.P.) using the revised Cochrane Risk-of-Bias tool for Randomized Trials (ROB 2)^46^ and the Risk of Bias in Non-randomized Studies–of Interventions (ROBINS-I).^47^ The ROB 2 tool was used to critically appraise the quality of the included RCTs in terms of randomness and concealment of participant allocation blinding from participants and outcome assessors, the proportion of missing data, selective reporting, and other sources of bias. Overall ROB was categorized as low risk, some concerns, or high risk. The ROBINS-I was used to critically appraise the quality of the included nonrandomized studies in terms of selection bias, confounding, and measurement bias, with the overall ROB judgment classified as low risk, moderate risk, serious risk, or critical risk. A third reviewer (L.L.K.H.) was consulted in case of disagreement.

### Statistical Analysis

If 2 or more similar controlled trails in terms of population and intervention were available for each outcome, their results were pooled by meta-analysis. Effect estimates of continuous outcomes were calculated using the SMDs and their 95% CIs. A random-effects model was used across all studies for data pooling. Heterogeneity of intervention effects among studies was quantified using the *I*^2^ statistic. Low variation was defined as *I*^2^ = 25%; moderate, *I*^2^ = 50%; and high, *I*^2^ = 75%.^48^ The effect size (SMD) was considered small with an SMD of 0.2 to 0.49; moderate, SMD of 0.5 to 0.79; and large, SMD of 0.8 or greater.^49^ The meta-analysis was conducted using Review Manager software version 5.4.1. (Cochrane Collaboration). The findings of studies that were not comparable and could not be included in the statistical pooling were summarized narratively. The findings of studies that were ineligible for meta-analysis because of inadequate data or profound heterogeneity in study design were summarized according to the Synthesis Without Meta-analysis (SWIM) reporting guideline.^50^ *P* values were 2-sided, and statistical significance was set at *P* = .05. Data were analyzed from July 10 to July 20, 2023.

## Results

### Systematic Review

A total of 24 studies^51,52,53,54,55,56,57,58,59,60,61,62,63,64,65,66,67,68,69,70,71,72,73,74^ with 717 participants (mean age range, 5.3 to 17.8 years; 141 [21.9%] female) were included. The search process and results are presented in the Figure. In total, 2580 records in English were retrieved, from which 674 duplicates were excluded. The titles and abstracts of the remaining 1906 records were screened; 179 were retained for full-text screening, of which 143 studies were excluded. After assessing the eligibility, 24 studies^51,52,53,54,55,56,57,58,59,60,61,62,63,64,65,66,67,68,69,70,71,72,73,74^ were included.

**Figure.**
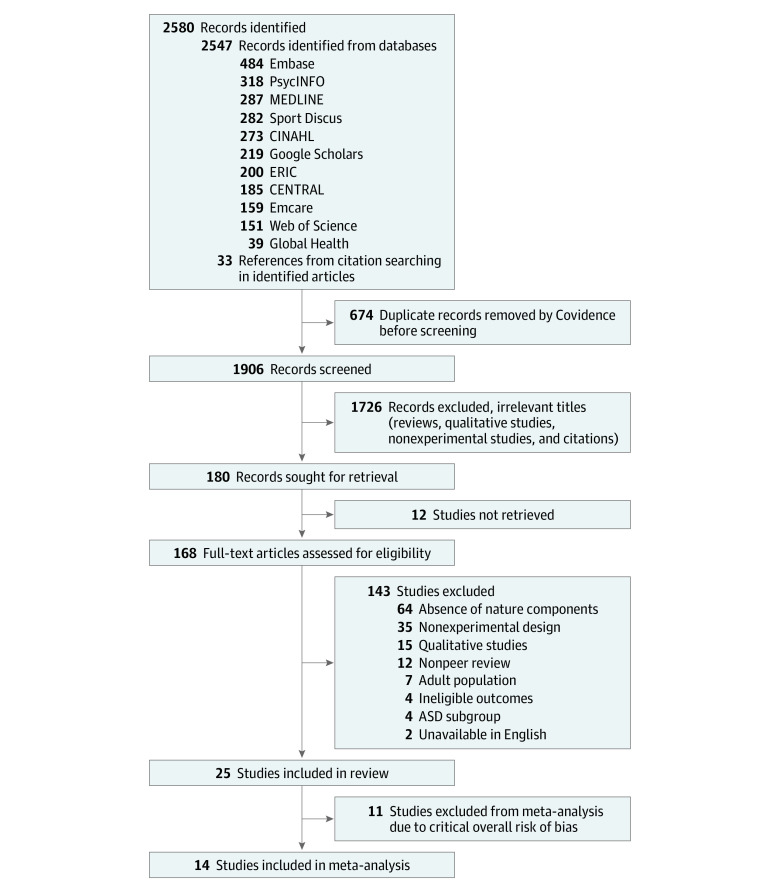
Flowchart for Study Retrieval and Selection ASD indicates autism spectrum disorder; CINAHL, Cumulative Index to Nursing and Allied Health Literature; ERIC, Education Resources Information Center.

The 24 studies included 7 RCTs,^68,69,70,71,72,73,74^ 6 quasi-experimental studies with control groups,^62,63,64,65,66,67^ and 11 single-group before-and-after studies^51,52,53,54,55,56,57,58,59,60,61^ (eTable 13 in Supplement 1). The included studies were published in English within the last 16 years (2006 to 2022); 12 studies^51,53,55,56,60,61,64,68,70,71,72,73^ were from the United States, 2 studies^52,63^ were from the United Kingdom, 2 studies^59,69^ were from Italy, 2 studies^54,65^ were from Iran, and there was 1 study each from Brazil,^74^ Canada,^57^ China,^67^ India,^58^ Israel,^66^ and Spain,^62^ with sample sizes ranging from 4 to 116 participants. No adverse effects were reported from included studies. The attrition rate of participants ranged from 0% to 41.5%. The key characteristics of the interventions are presented in eTable 13 in Supplement 1. The contents of the NBIs varied, although 20 studies (83%) reported recreational therapy focused on equine-assisted activities,^52,54,56,61,62,63,64,67,68,70,71,72^ equine-assisted therapy,^51,69,73,74^ adventure activities,^66^ surfing,^53^ golfing,^60^ and summer camp^55^; the remaining 4 studies (17%) included nature therapy,^65^ horticultural therapy,^59^ outdoor music therapy,^57^ and outdoor art therapy.^58^ These interventions were all under the same category of rehabilitation based on the National Library of Medicine Medical Subject Headings.

All interventions were face-to-face and administered to children with ASD in natural outdoor settings. Regarding the teaching and learning modality, all included studies reflected experiential learning. A group-based mode was adopted for intervention delivery in 22 studies.^51,52,53,54,55,56,57,58,59,60,61,62,63,64,66,67,68,69,70,71,72,74^ In 14 studies,^51,52,60,61,62,63,64,67,68,69,70,71,73,74^ the intervenors were qualified instructors. The presence of protocols or standardized treatment manuals to guarantee fidelity was noted in 4 studies.^71,72,73,74^ The assessment of treatment integrity by a checklist developed to ensure adherence to protocols was only indicated in 4 studies.^63,71,72,73^ There were 3 studies^71,72,73^ that reported numeric data for mean fidelity rating. The characteristics of the interventions are presented in eTable 14 in Supplement 1.

Data on socioeconomic status and funding sources in the included studies were extracted. Reporting of this information was inconsistent, with only 2 studies^54,73^ providing data on socioeconomic status. Funding was reported by 6 studies^56,59,62,70,71,73^ (25%), while 2 studies^65,67^ (8%) explicitly mentioned no funding. A large proportion of studies (16 studies^49,51,52,53,54,55,57,60,61,63,64,66,68,69,72,74^ [67%]) did not provide funding status. Based on reported funding sources, a direct association between funding and the barrier to access cannot be established.

### Risk of Bias

All 7 included RCTs^68,69,70,71,72,73,74^ were classified as having some ROB concerns (eFigure 1 in Supplement 1). The lack of allocation concealment was noted in all RCTs. Among 17 non-RCT studies,^51,52,53,54,55,56,57,58,59,60,61,62,63,64,65,66,67^ 3 studies^62,66,67^ were classified as having low overall ROB and 3 studies^63,64,65^ were noted as having moderate overall ROB. Critical overall ROB was noted in 11 studies^51,52,53,54,55,56,57,58,59,60,61^ using the ROBIN-I tool (eFigure 2 in Supplement 1). The outcomes from studies with critical overall bias were excluded from the meta-analysis.

### Meta-Analyses of Health-Related Functioning Outcomes

We summarized the study outcomes and measurements of the 24 included studies^51,52,53,54,55,56,57,58,59,60,61,62,63,64,65,66,67,68,69,70,71,72,73,74^ (eTable 15 in Supplement 1). We assessed 13 studies using the random-effects model for meta-analysis, the synthesized SMDs, and their 95% CIs for the outcomes immediately after the intervention (Table). Among the social functioning outcomes, the pooled estimates indicated a significant moderate association with social communication (SMD, −0.59; 95% CI, −0.85 to −0.34; *P* < .001; *I*^2^ = 0%). Significant small to moderate associations were observed for autistic mannerisms (SMD, −0.47; 95% CI, −0.89 to −0.06; *P* = .02; *I*^2^ = 51%), social cognition (SMD, −0.45; 95% CI, −0.69 to −0.22; *P* < .001; *I*^2^ = 0%), social motivation (SMD, −0.40; 95% CI, −0.64 to −0.17; *P* < .001; *I*^2^ = 0%), and social awareness (SMD, −0.31; 95% CI, −0.57 to −0.06; *P* = .02; *I*^2^ = 10%). The pooled estimates for behavioral functioning outcomes indicated a significant moderate association with reduced hyperactivity (SMD, −0.56; 95% CI, −0.86 to −0.26; *P* < .001; *I*^2^ = 0%) and a small to moderate association with reduced irritability (SMD, −0.49; 95% CI, −0.79 to −0.19; *P* = .001; *I*^2^ = 0%). The pooled estimates for sensory functioning outcomes showed a significant large association with improved inattention and distractibility (SMD, 1.13; 95% CI, 0.67 to 1.60; *P* < .001; *I*^2^ = 0%). Significant moderate associations were observed for sensory seeking (SMD, 0.77; 95% CI, 0.33 to 1.22; *P* < .001; *I*^2^ = 0%) and sensory sensitivity (SMD, 0.56; 95% CI, 0.12 to 1.00; *P* = .01; *I*^2^ = 0%). No significant association was found with improvement in speech and language.

**Table.** Summary of Meta-Analysis Results on Each Postintervention Outcome

zOutcome	Studies, No.	SMD (95% CI)	*t*	*P* value	*I*^2^, %
Autistic mannerism	4	−0.47 (−0.89 to −0.06)	2.24	.02	51
Daily living skills	3	0.08 (−0.49 to 0.65)	0.28	.78	51
Fine motor and perception	2	0.39 (−0.04 to 0.83)	1.78	.07	0
Hyperactivity	4	−0.56 (−0.86 to −0.26)	3.63	<.001	0
Inattention and distractibility	2	1.13 (0.67 to 1.60)	4.77	<.001	0
Inappropriate speech	3	−0.20 (−0.51 to 0.11)	1.24	.21	0
Irritability	4	−0.49 (−0.79 to −0.19)	3.21	.001	0
Lethargy	2	−0.61 (−1.58 to 0.36)	1.23	.22	67
Number of different words	2	0.17 (−0.46 to 0.80)	0.53	.59	41
Number of words	2	0.22 (−0.64 to 1.09)	0.51	.61	63
Sedentary	2	0.70 (−0.10 to 1.49)	1.72	.09	66
Sensory seeking	2	0.77 (0.33 to 1.22)	3.40	<.001	0
Sensory sensitivity	2	0.56 (0.12 to 1.00)	2.50	.01	0
Social awareness	6	−0.31 (−0.57 to −0.06)	2.41	.02	10
Social cognition	6	−0.45 (−0.69 to −0.22)	3.77	<.001	0
Social communication	5	−0.59 (−0.85 to −0.34)	4.58	<.001	0
Social motivation	6	−0.40 (−0.64 to −0.17)	3.35	<.001	0
Socialization	3	−0.12 (−0.79 to 0.54)	0.37	.71	62
Stereotype	3	−0.05 (−0.36 to 0.27)	0.29	.77	0

Abbreviation: SMD, standardized mean difference.

### Syntheses Without Meta-Analysis of Health-Related Subjective Well-Being

Only 2 studies examined the association between NBIs involving horses and quality of life (QOL), including equine-assisted therapy^62^ and equine-assisted activity.^64^ Both studies indicated a positive association between NBIs and specific subdomains of QOL. Two studies^73,74^ assessed disabilities, but neither found a significant association between the equine-assisted intervention and the level of disability. A significant association was suggested between equine-assisted therapy and ASD severity using the Childhood Autism Rating Scale (CARS) in 2 studies.^63,74^ Lastly, a positive association was reported between equine-assisted therapy and goal attainment,^73^ as well as between nature therapy and the parent-children relationship.^65^

## Discussion

This systematic review and meta-analysis introduced the concept of expanding the natural environment to encompass community settings by classifying health-related outcomes of NBIs in children with ASD, with reference to the WHO ICF,^75^ to provide a holistic perspective on overall health. To ensure a comprehensive search and given the limited literature on NBIs in experimental designs, specific health-related outcomes were not predetermined prior to data extraction. Functional outcomes included behavioral, sensory, emotional, and social functioning, providing insights into the abilities, skills, and daily performance of children with ASD, consistent with *DSM-5* criteria. Outcomes outside functional categories are subclassified under subjective well-being, and these are all directly related to the unique challenges and experiences of children with ASD.

Our systematic review and meta-analysis suggested a favorable association of NBIs with improvements in all functional outcomes except emotional functioning. Of 13 studies included in meta-analysis, 10 reported associations between NBIs and sensory,^68,70,71^ social,^66,67,68,70,71,72,73^ and behavioral^62,63,66,69,71,72,73^ functioning. Significant improvement was observed in sensory functioning outcomes, particularly in the ability to sustain attention. Sensory-based interventions in conventional studies are usually conducted in clinic or education settings.^2,76^ This systematic review and meta-analysis is the first study to our knowledge to suggest an alternative approach to improve sensory functioning among children with ASD using NBIs outside of traditional settings. Notable associations were found between NBIs and improved hyperactivity and irritability. Previous literature often addressed these outcomes through behavioral interventions, developmental interventions, naturalistic development behavioral interventions, sensory-based interventions, cognitive behavioral therapy, and Treatment and Education of Autistic and Related Communication-Handicapped Children.^5^ Social functioning was the most common outcome addressed in psychosocial interventions.^3^ This systematic review and meta-analysis also observed a significant association between NBIs and social functioning. However, the association between NBIs and subjective well-being could not be determined due to limited evidence.

The *DSM-5* emphasizes considerations of an individual’s functioning and overall well-being, in the form of their strengths and weaknesses.^77^ However, the dimensional nature of ASD in *DSM-5* challenges the linear assessment of severity.^78^ When examining the association between NBIs and ASD severity, it is recommended to supplement the CARS with other tools aligned with the spectrum approach in *DSM-5*, although the CARS can provide insights into specific ASD-related behavioral characteristics. Likewise, caution is needed when using *DSM-5* criteria to assess the level of disability in children with ASD because autism is not inherently a disability. Relying solely on this factor as an outcome measure may inadvertently ignore neurodiversity. Adopting the suggested health-related outcome classification used this systematic review and meta-analysis may allow for a holistic approach to evaluating interventions while embracing the diverse experiences and strengths of individuals with ASD.

### Implications for Research

This systematic review and meta-analysis provides insights to guide treatment decisions and interventions for children with ASD. It identifies areas where NBIs could positively aid their trajectory. Although the aspect of emotional functioning remains uncertain, previous reviews in relevant populations have shown positive outcomes in emotional functioning and subjective well-being associated with NBIs. These outcomes include improved psychological well-being,^14^ enhanced self-esteem and resilience, overall well-being,^37^ and mental health.^38^ It is recommended that future research continue to address this outcome gap.

It is important to note that the overall quality of evidence supporting NBIs is limited. This highlights the need for future research to strengthen the evidence base through robust research designs. Furthermore, our systematic review and meta-analysis highlights the importance of follow-up assessments at longer time points after the completion of an intervention to assess the sustainability of improvements in health-related outcomes. Additionally, fidelity and treatment integrity should be carefully considered in future studies to ensure the validity, reliability, and generalizability of findings and to facilitate the translation of research into practice. It is also important for future studies to consider the prevalent comorbidities among individuals with ASD,^79^ as are relevant to the outcomes identified in this systematic review and meta-analysis. Finally, the cost of NBIs may hinder access. Future studies are recommended to examine the specific costs associated with different types of NBIs and their impacts on accessibility.

### Limitations

This systematic review and meta-analysis has several limitations. First, it is important to recognize that the findings may not fully represent the outcomes associated with all NBIs. The meta-analysis focused primarily on recreational therapy studies, excluding other types of interventions, such as horticultural therapy, outdoor art, and music therapy, due to methodological flaws. On the other hand, although all included studies were classified as recreational therapy, there was a wide range of interventions in this category. Recognizing the limitations of subgroup analysis is critical to understanding the overall effects of different NBIs and considering potential variations in their effectiveness. Second, issues were identified in assessing ROB, including incomplete information on allocation concealment, unblinding of participants and intervenors, and outcome measures. Therefore, caution should be exercised when interpreting the findings of this review. Third, all included studies lacked a theoretical framework. Despite the early stage of developing NBIs for children with ASD, it is urged to use theoretical or conceptual frameworks to guide study designs and ensure results are interpretable and generalizable. Fourth, language and publication biases may have affected the findings of this review. Studies in non–English languages were excluded, limiting our understanding of NBIs outside the Western context. Additionally, publication bias (eg, positive studies are more likely to be published) may affect the meta-analysis if null or negative studies are not published. Cultural context also influences perceptions of NBIs, and further studies are needed to explore these perceptions among children with ASD and their caregivers in non-Western contexts.

## Conclusions

The findings of this systematic review and meta-analysis suggest that NBIs in the form of group-based recreational therapy with experiential learning were associated with improved short-term sensory, social, and behavioral functioning outcomes among children with ASD. More empirical evidence is required regarding the emotional functioning and subjective well-being outcomes using robust study design to aid the functional and health trajectories of children with ASD.

## Notes

Supplement 1. **eTable 1.** Population, Intervention, Comparison, and Outcome (PICO) Table of Study Eligibility Criteria **eTable 2.** Study Search Strategy **eTable 3.** CINAHL Search Results **eTable 4.** Cochrane Search Results **eTable 5.** Embase Search Results **eTable 6.** Emcare Search Results **eTable 7.** ERIC Search Results **eTable 8.** Global Health Search Results **eTable 9.** Medline Search Results **eTable 10.** PsycInfo Search Results **eTable 11.** SPORTDiscus Search Results **eTable 12.** Web of Science Search Results **eTable 13.** Summary Description of the Characteristics of Included Studies **eTable 14.** Summary Description of the Intervention of Included Studies **eFigure 1.** Revised Cochrane Risk-of-Bias Tool for Randomized Trials Summary and Author Judgments of Low, Some Concerns, and High Risk of Bias Across All Included RCTs **eFigure 2.** Cochrane Risk of Bias in Nonrandomized Studies of Intervention (ROBINS-I) Summary and Author Judgments of Low, Moderate, Serious, and Critical Risk of Bias Across All Included Non-RCTs **eTable 15.** Summary Description of the Outcomes and Measurements of Included Studies

Supplement 2.
Data Sharing Statement
