# Supplementary material for: Nature-Based Interventions for Autistic Children: A Systematic Review and Meta-Analysis
Source: JAMA Netw Open. 2023 Dec 7;6(12):e2346715. doi: 10.1001/jamanetworkopen.2023.46715 (PMC10704280; doi:10.1001/jamanetworkopen.2023.46715)
Supplement: Supplement 1. — eTable 1. Population, Intervention, Comparison, and Outcome (PICO) Table of Study Eligibility Criteria eTable 2. Study Search Strategy eTable 3. CINAHL Search Results eTable 4. Cochrane Search Results eTable 5. Embase Search Results eTable 6. Emcare Search Results eTable 7. ERIC Search Results eTable 8. Global Health Search Results eTable 9. Medline Search Results eTable 10. PsycInfo Search Results eTable 11. SPORTDiscus Search Results eTable 12. Web of Science Search Results eTable 13. Summary Description of the Characteristics of Included Studies eTable 14. Summary Description of the Intervention of Included Studies eFigure 1. Revised Cochrane Risk-of-Bias Tool for Randomized Trials Summary and Author Judgments of Low, Some Concerns, and High Risk of Bias Across All Included RCTs eFigure 2. Cochrane Risk of Bias in Nonrandomized Studies of Intervention (ROBINS-I) Summary and Author Judgments of Low, Moderate, Serious, and Critical Risk of Bias Across All Included Non-RCTs eTable 15. Summary Description of the Outcomes and Measurements of Included Studies [file jamanetwopen-e2346715-s001.pdf]

Supplemental Online Content

Fan MSN, Li WHC, Ho LLK, Phiri L, Choi KC. Nature-based interventions for autistic children. *JAMA Netw Open.* 2023;6(12):e2346715. doi:10.1001/jamanetworkopen.2023.46715

eTable 1. Population, Intervention, Comparison, and Outcome (PICO) Table of Study Eligibility Criteria.
eTable 2. Study Search Strategy
eTable 3. CINAHL Search Results
eTable 4. Cochrane Search Results
eTable 5. Embase Search Results
eTable 6. Emcare Search Results
eTable 7. ERIC Search Results
eTable 8. Global Health Search Results
eTable 9. Medline Search Results
eTable 10. PsycInfo Search Results
eTable 11. SPORTDiscus Search Results
eTable 12. Web of Science Search Results
eTable 13. Summary Description of the Characteristics of Included Studies
eTable 14. Summary Description of the Intervention of Included Studies
eFigure 1. Revised Cochrane Risk-of-Bias Tool for Randomized Trials Summary and Author Judgments of Low, Some Concerns, and High Risk of Bias Across All Included RCTs
eFigure 2. Cochrane Risk of Bias in Nonrandomized Studies of Intervention (ROBINS-I) Summary and Author Judgments of Low, Moderate, Serious, and Critical Risk of Bias Across All Included Non-RCTs
eTable 15. Summary Description of the Outcomes and Measurements of Included Studies

This supplemental material has been provided by the authors to give readers additional information about their work.

© 2023 Fan MSN et al. *JAMA Network Open.*

eTable 1. Population, intervention, Comparison, and Outcome (PICO) Table of Study Eligibility Criteria.

**P**	**Population**<br>Children under 18 years old diagnosed with ASD, defined in DSM-5 or ICD-11 criteria, with or without mental and/or physical health problems, were considered for inclusion. In addition, children diagnosed with pervasive developmental disorders, as defined in ICD-10 criteria or in previous versions of DSM, including childhood autism, atypical autism, Asperger's syndrome, and pervasive developmental disorder not otherwise specified, as these previous diagnostic labels are now included in the category of ASD in DSM-5 and ICD-11. Children with the diagnostic label of Rett's disorder or childhood disintegrative disorder were omitted since they are excluded from the ASD diagnostic category in the current classification.
**I**	**Interventions**<br>Interventions in the form of independent or group-based activities in the natural outdoor settings are referred to as outdoor green or blue spaces. Physical activities undertaken outdoors where nature was not an essential component were considered ineligible.
**C**	**Comparators**<br>Interventions that are compared with either placebo, waitlist control, or standard care control were included in experimental studies.
**O**	**Outcome measures**<br>The health-related outcomes had to be measured in at least one of the primary outcomes or one of the secondary outcomes reported in the section on data collection items. Non-standardized or standardized instruments could be included with either self-report or observation measures. Non-patient-centric biochemical outcomes were excluded.

© 2023 Fan MSN et al. *JAMA Network Open.*

eTable 2. Study Search Strategy

Search Concepts	MeSH	Keywords (add all possible synonyms, variations, and related terms)
Health-related outcomes		Health?related outcome* OR Communication* OR cooperation OR decision making OR equity OR health* OR well?being OR independent* OR flexib* OR interpersonal OR life?skill* OR personal development OR pro?social* OR pro?social behavior* OR resilien* OR psycho?social OR self?concept OR self?confiden* OR self?esteem OR soci?emotion* OR team?work OR Quality of life OR self?efficacy OR psycho*
Nature-based		(Nature?based* OR recreation* OR environment* OR outdoor OR natur* OR natur* environment* OR natur* connect* OR natur* experience* OR natur* therap* OR ecotheap* OR ecopsychosocial* OR life style* OR green* OR out?door play* OR Park* OR greenspace* OR green space* OR ocean* OR bluespace* OR blue space* OR surf* OR sail* OR voyage* OR forest* OR forest school* OR garden* OR golf* OR biodiverse* OR Horticultur* OR leisure* OR wild* OR adventure* OR Canine therap* OR Equine therap* OR Hippotherap* OR Horse rid* OR Adventur* therap* OR wilderness* OR camp* OR summer camp* OR out?patient* OR communit* OR experiential?learning) NOT (pharmacological* OR pharmaceutical*)
Intervention	Methods/	Intervention* OR therap* OR teach* OR program* OR package* OR support* OR strateg* OR service* OR learning*
Children	Child/	Child* OR youth* OR adolesc* OR teen*
Autism spectrum disorder (ASD)	Autism Spectrum Disorder/ OR Autistic Disorder/ OR Asperger Syndrome/	(autis* OR high function* autis* OR Asperger* OR pervasive development disorder* OR neurodevelopmental disorder) NOT (ADHD OR attention deficit hyperactivity disorder)
		(Randomized controlled trial OR case control study OR experimental* OR quasi?experimental* OR controlled clinical trial OR randomized clinical trials OR randomi* OR random allocation OR double blind* method OR single blind* method) NOT (protocol* OR guideline* OR procedure* OR polic* OR review OR meta analysis OR systematic review)

© 2023 Fan MSN et al. *JAMA Network Open.*

eTable 3. CINAHL Search Results

EBSCOhost

Thursday, April 27, 2023 3:58:09 AM

#	Query	Limiters/Expanders	Last Run Via	Results
S11	S9 AND S10	Expanders - Apply equivalent subjects<br>Search modes - Boolean/Phrase	Interface - EBSCOhost Research Databases<br>Search Screen - Advanced Search<br>Database - CINAHL Ultimate	273
S10	(Randomized controlled trial OR case control study OR experimental* OR quasi? experimental* OR controlled clinical trial OR randomized clinical trials OR randomi* OR random allocation OR double blind* method OR single blind* method) NOT (protocol* OR guideline* OR procedure* OR polic* OR review OR meta analysis OR systematic review)	Expanders - Apply equivalent subjects<br>Search modes - Boolean/Phrase	Interface - EBSCOhost Research Databases<br>Search Screen - Advanced Search<br>Database - SPORTDiscus with Full Text	66,231
S9	S5 AND S8	Expanders - Apply equivalent subjects<br>Search modes - Boolean/Phrase	Interface - EBSCOhost Research Databases<br>Search Screen - Advanced Search<br>Database - SPORTDiscus with Full Text	282
S8	S6 AND S7	Expanders - Apply equivalent subjects<br>Search modes - Boolean/Phrase	Interface - EBSCOhost Research Databases<br>Search Screen - Advanced Search<br>Database - SPORTDiscus with Full Text	1,460
S7	(Autism Spectrum Disorder/ OR Autistic Disorder/ OR Asperger Syndrome/) OR ((autis* OR high function* autis* OR Asperger* OR pervasive development disorder* OR neurodevelopmental disorder)) NOT ((ADHD OR attention deficit hyperactivity disorder))	Expanders - Apply equivalent subjects<br>Search modes - Boolean/Phrase	Interface - EBSCOhost Research Databases<br>Search Screen - Advanced Search<br>Database - SPORTDiscus with Full Text	2,191
S6	Child/ OR (Child* OR youth* OR adolesc* OR teen*)	Expanders - Apply equivalent subjects<br>Search modes - Boolean/Phrase	Interface - EBSCOhost Research Databases<br>Search Screen - Advanced Search<br>Database - SPORTDiscus with Full Text	193,593
S5	S1 AND S4	Expanders - Apply equivalent subjects<br>Search modes - Boolean/Phrase	Interface - EBSCOhost Research Databases<br>Search Screen - Advanced Search<br>Database - SPORTDiscus with Full Text	117,453
S4	S2 AND S3	Expanders - Apply equivalent subjects<br>Search modes - Boolean/Phrase	Interface - EBSCOhost Research Databases<br>Search Screen - Advanced Search<br>Database - SPORTDiscus with Full Text	214,638
S3	Methods/ OR (Intervention* OR therap* OR teach* OR program* OR package* OR support* OR strateg* OR service* OR learning*)	Expanders - Apply equivalent subjects<br>Search modes - Boolean/Phrase	Interface - EBSCOhost Research Databases<br>Search Screen - Advanced Search<br>Database - SPORTDiscus with Full Text	776,941
S2	((Nature?based* OR recreation* OR environment* OR outdoor OR natur* OR natur* environment* OR natur* connect* OR natur* experience* OR natur* therap* OR ecotheap* OR ecopsychosocial* OR life style* OR green* OR out?door play* OR Park* OR greenspace* OR green space* OR ocean* OR bluespace* OR blue space* OR surf* OR sail* OR voyage* OR forest* OR forest school* OR garden* OR golf* OR biodiverse* OR Horticultur* OR leisure* OR wild* OR adventure* OR Canine therap* OR Equine therap* OR Hippotherap* OR Horse rid* OR Adventur* therap* OR wilderness* OR camp* OR summer camp* OR out?patient* OR communit* OR experiential?learning)) NOT ((pharmacological* OR pharmaceutical*))	Expanders - Apply equivalent subjects<br>Search modes - Boolean/Phrase	Interface - EBSCOhost Research Databases<br>Search Screen - Advanced Search<br>Database - SPORTDiscus with Full Text	616,674
S1	Health?related outcome* OR Communication* OR cooperation OR decision making OR equity OR health* OR well?being OR independent* OR flexib* OR interpersonal OR life?skill* OR personal development OR pro?social* OR pro?social behavior* OR resilien* OR psycho?social OR self?concept OR self? confiden* OR self?esteem OR soci? emotion* OR team?work OR Quality of life OR self?efficacy OR psycho*	Expanders - Apply equivalent subjects<br>Search modes - Boolean/Phrase	Interface - EBSCOhost Research Databases<br>Search Screen - Advanced Search<br>Database - SPORTDiscus with Full Text	651,260

© 2023 Fan MSN et al. *JAMA Network Open*.

eTable 4. Cochrane Search Results

Date Run: 27/04/2023 06:03:26

ID	Search Hits	
#1	Health?related outcome* OR Communication* OR cooperation OR decision making OR equity OR health* OR well?being OR independent* OR flexib* OR interpersonal OR life?skill* OR personal development OR pro?social* OR pro?social behavior* OR resilien* OR psycho?social OR self?concept OR self?confiden* OR self?esteem OR soci?emotion* OR team?work OR Quality of life OR self?efficacy OR psycho*	770675
#2	(Nature?based* OR recreation* OR environment* OR outdoor OR natur* OR natur* environment* OR natur* connect* OR natur* experience* OR natur* therap* OR ecotheap* OR ecopsychosocial* OR life style* OR green* OR out?door play* OR Park* OR greenspace* OR green space* OR ocean* OR bluespace* OR blue space* OR surf* OR sail* OR voyage* OR forest* OR forest school* OR garden* OR golf* OR biodiverse* OR Horticultur* OR leisure* OR wild* OR adventure* OR Canine therap* OR Equine therap* OR Hippotherap* OR Horse rid* OR Adventur* therap* OR wilderness* OR camp* OR summer camp* OR out?patient* OR communit* OR experiential?learning) NOT (pharmacological* OR pharmaceutical*)	305272
#3	Methods OR Intervention* OR therap* OR teach* OR program* OR package* OR support* OR strateg* OR service* OR learning	1527307
#4	(Child* OR youth* OR adolesc* OR teen*) AND (autis* OR high function* autis* OR Asperger* OR pervasive development disorder* OR neurodevelopmental disorder) NOT (ADHD OR attention deficit hyperactivity disorder)	4840
#5	#1 AND #2 AND #3 AND #4 with 'Complementary Medicine', 'Common Mental Disorders', 'Child Health', 'Developmental, Psychosocial and Learning Problems' in Cochrane Groups	221

© 2023 Fan MSN et al. *JAMA Network Open.*

eTable 5. Embase Search Results

EMBASE via Ovid, Embase 1910 to Present

1	(Health?related outcome* or Communication* or cooperation or decision making or equity or health* or well?being or independent* or flexib* or interpersonal or life?skill* or personal development or pro?social* or pro?social behavior* or resilien* or psycho?social or self?concept or self?confiden* or self?esteem or soci?emotion* or team?work or Quality of life or self?efficacy or psycho*).mp. [mp=title, abstract, heading word, drug trade name, original title, device manufacturer, drug manufacturer, device trade name, keyword heading word, floating subheading word, candidate term word]	10348560
2	((Nature?based* or recreation* or environment* or outdoor or natur* or natur* environment* or natur* connect* or natur* experience* or natur* therap* or ecotheap* or ecopsychosocial* or life style* or green* or out?door play* or Park* or greenspace* or green space* or ocean* or bluespace* or blue space* or surf* or sail* or voyage* or forest* or forest school* or garden* or golf* or biodiverse* or Horticultur* or leisure* or wild* or adventure* or Canine therap* or Equine therap* or Hippotherap* or Horse rid* or Adventur* therap* or wilderness* or camp* or summer camp* or out?patient* or communit* or experiential?learning) not (pharmacological* or pharmaceutical*)).mp. [mp=title, abstract, heading word, drug trade name, original title, device manufacturer, drug manufacturer, device trade name, keyword heading word, floating subheading word, candidate term word]	7257881
3	Methods/ or Intervention*.mp. or therap*.mp. or teach*.mp. or program*.mp. or package*.mp. or support*.mp. or strateg*.mp. or service*.mp. or learning*.mp. [mp=title, abstract, heading word, drug trade name, original title, device manufacturer, drug manufacturer, device trade name, keyword heading word, floating subheading word, candidate term word]	16159569
4	2 and 3	2980582
5	1 and 4	1303206
6	Child/ or Child*.mp. or youth*.mp. or adolesc*.mp. or teen*.mp. [mp=title, abstract, heading word, drug trade name, original title, device manufacturer, drug manufacturer, device trade name, keyword heading word, floating subheading word, candidate term word]	4014542
7	Autism Spectrum Disorder/ or Autistic Disorder/ or Asperger Syndrome/	68257
8	((autis* or high function* autis* or Asperger* or pervasive development disorder* or neurodevelopmental disorder) not (ADHD or attention deficit hyperactivity disorder)).mp. [mp=title, abstract, heading word, drug trade name, original title, device manufacturer, drug manufacturer, device trade name, keyword heading word, floating subheading word, candidate term word]	98994
9	7 or 8	104843
10	6 and 9	62089
11	5 and 10	6136
12	((Randomized controlled trial or case control study or experimental* or quasi?experimental* or controlled clinical trial or randomized clinical trials or randomi* or random allocation or double blind* method or single blind* method) not (protocol* or guideline* or procedure* or polic* or review or meta analysis or systematic review)).mp. [mp=title, abstract, heading word, drug trade name, original title, device manufacturer, drug manufacturer, device trade name, keyword heading word, floating subheading word, candidate term word]	2327547
13	11 and 12	434

© 2023 Fan MSN et al. *JAMA Network Open*.

eTable 6. Emcare Search Results

EMCARE via Ovid, Ovid Emcare <1995 to 2023 Week 16>

1	(Health?related outcome* or Communication* or cooperation or decision making or equity or health* or well?being or independent* or flexib* or interpersonal or life?skill* or personal development or pro?social* or pro?social behavior* or resilien* or psycho?social or self?concept or self?confiden* or self?esteem or soci?emotion* or team?work or Quality of life or self?efficacy or psycho*).mp. [mp=title, abstract, heading word, drug trade name, original title, device manufacturer, drug manufacturer, device trade name, keyword heading word]	2985278
2	((Nature?based* or recreation* or environment* or outdoor or natur* or natur* environment* or natur* connect* or natur* experience* or natur* therap* or ecotheap* or ecopsychosocial* or life style* or green* or out?door play* or Park* or greenspace* or green space* or ocean* or bluespace* or blue space* or surf* or sail* or voyage* or forest* or forest school* or garden* or golf* or biodiverse* or Horticultur* or leisure* or wild* or adventure* or Canine therap* or Equine therap* or Hippotherap* or Horse rid* or Adventur* therap* or wilderness* or camp* or summer camp* or out?patient* or communit* or experiential?learning) not (pharmacological* or pharmaceutical*)).mp. [mp=title, abstract, heading word, drug trade name, original title, device manufacturer, drug manufacturer, device trade name, keyword heading word]	1440428
3	Methods/ or Intervention*.mp. or therap*.mp. or teach*.mp. or program*.mp. or package*.mp. or support*.mp. or strateg*.mp. or service*.mp. or learning*.mp. [mp=title, abstract, heading word, drug trade name, original title, device manufacturer, drug manufacturer, device trade name, keyword heading word]	3200590
4	2 and 3	705652
5	1 and 4	431691
6	Child/ or Child*.mp. or youth*.mp. or adolesc*.mp. or teen*.mp. [mp=title, abstract, heading word, drug trade name, original title, device manufacturer, drug manufacturer, device trade name, keyword heading word]	1006285
7	Autism Spectrum Disorder/ or Autistic Disorder/ or Asperger Syndrome/	16660
8	((autis* or high function* autis* or Asperger* or pervasive development disorder* or neurodevelopmental disorder) not (ADHD or attention deficit hyperactivity disorder)).mp. [mp=title, abstract, heading word, drug trade name, original title, device manufacturer, drug manufacturer, device trade name, keyword heading word]	35994
9	7 or 8	36842
10	6 and 9	23685
11	5 and 10	2263
12	((Randomized controlled trial or case control study or experimental* or quasi?experimental* or controlled clinical trial or randomized clinical trials or randomi* or random allocation or double blind* method or single blind* method) not (protocol* or guideline* or procedure* or polic* or review or meta analysis or systematic review)).mp. [mp=title, abstract, heading word, drug trade name, original title, device manufacturer, drug manufacturer, device trade name, keyword heading word]	468266
13	11 and 12	159

© 2023 Fan MSN et al. *JAMA Network Open.*

eTable 7. ERIC Search Results

EBSCOhost

Thursday, April 27, 2023 4:00:07 AM

#	Query	Limiters/Expanders	Last Run Via	Results
S11	S9 AND S10	Expanders - Apply equivalent subjects Search modes - Boolean/Phrase	Interface - EBSCOhost Research Databases Search Screen - Advanced Search Database - ERIC	200
S10	(Randomized controlled trial OR case control study OR experimental* OR quasi?experimental* OR controlled clinical trial OR randomized clinical trials OR randomi* OR random allocation OR double blind* method OR single blind* method) NOT (protocol* OR guideline* OR procedure* OR polic* OR review OR meta analysis OR systematic review)	Expanders - Apply equivalent subjects Search modes - Boolean/Phrase	Interface - EBSCOhost Research Databases Search Screen - Advanced Search Database - SPORTDiscus with Full Text	66,231
S9	S5 AND S8	Expanders - Apply equivalent subjects Search modes - Boolean/Phrase	Interface - EBSCOhost Research Databases Search Screen - Advanced Search Database - SPORTDiscus with Full Text	282
S8	S6 AND S7	Expanders - Apply equivalent subjects Search modes - Boolean/Phrase	Interface - EBSCOhost Research Databases Search Screen - Advanced Search Database - SPORTDiscus with Full Text	1,460
S7	(Autism Spectrum Disorder/ OR Autistic Disorder/ OR Asperger Syndrome/) OR ((autis* OR high function* autis* OR Asperger* OR pervasive development disorder* OR neurodevelopmental disorder)) NOT ((ADHD OR attention deficit hyperactivity disorder))	Expanders - Apply equivalent subjects Search modes - Boolean/Phrase	Interface - EBSCOhost Research Databases Search Screen - Advanced Search Database - SPORTDiscus with Full Text	2,191
S6	Child/ OR (Child* OR youth* OR adolesc* OR teen*)	Expanders - Apply equivalent subjects Search modes - Boolean/Phrase	Interface - EBSCOhost Research Databases Search Screen - Advanced Search Database - SPORTDiscus with Full Text	193,593
S5	S1 AND S4	Expanders - Apply equivalent subjects Search modes - Boolean/Phrase	Interface - EBSCOhost Research Databases Search Screen - Advanced Search Database - SPORTDiscus with Full Text	117,453
S4	S2 AND S3	Expanders - Apply equivalent subjects Search modes - Boolean/Phrase	Interface - EBSCOhost Research Databases Search Screen - Advanced Search Database - SPORTDiscus with Full Text	214,638
S3	Methods/ OR (Intervention* OR therap* OR teach* OR program* OR package* OR support* OR strateg* OR service* OR learning*)	Expanders - Apply equivalent subjects Search modes - Boolean/Phrase	Interface - EBSCOhost Research Databases Search Screen - Advanced Search Database - SPORTDiscus with Full Text	776,941
S2	((Nature?based* OR recreation* OR environment* OR outdoor OR natur* OR natur* environment* OR natur* connect* OR natur* experience* OR natur* therap* OR ecotheap* OR ecopsychosocial* OR life style* OR green* OR out?door play* OR Park* OR greenspace* OR green space* OR ocean* OR bluespace* OR blue space* OR surf* OR sail* OR voyage* OR forest* OR forest school* OR garden* OR golf* OR biodiverse* OR Horticultur* OR leisure* OR wild* OR adventure* OR Canine therap* OR Equine therap* OR Hippotherap* OR Horse rid* OR Adventur* therap* OR wilderness* OR camp* OR summer camp* OR out?patient* OR communit* OR experiential?learning)) NOT ((pharmacological* OR pharmaceutical*))	Expanders - Apply equivalent subjects Search modes - Boolean/Phrase	Interface - EBSCOhost Research Databases Search Screen - Advanced Search Database - SPORTDiscus with Full Text	616,674
S1	Health?related outcome* OR Communication* OR cooperation OR decision making OR equity OR health* OR well?being OR independent* OR flexib* OR interpersonal OR life?skill* OR personal development OR pro?social* OR pro?social behavior* OR resilien* OR psycho?social OR self?concept OR self?confiden* OR self?esteem OR soci?emotion* OR team?work OR Quality of life OR self?efficacy OR psycho*	Expanders - Apply equivalent subjects Search modes - Boolean/Phrase	Interface - EBSCOhost Research Databases Search Screen - Advanced Search Database - SPORTDiscus with Full Text	651,260

© 2023 Fan MSN et al. *JAMA Network Open*.

Table 8. Global Health Search Results

Global Health via Ovid, Global Health <1973 to 2023 Week 16>

1	(Health?related outcome* or Communication* or cooperation or decision making or equity or health* or well?being or independent* or flexib* or interpersonal or life?skill* or personal development or pro?social* or pro?social behavior* or resilien* or psycho?social or self?concept or self?confiden* or self?esteem or soci?emotion* or team?work or Quality of life or self?efficacy or psycho*).mp. [mp=abstract, title, original title, heading words, cabicodes words]	1728782
2	((Nature?based* or recreation* or environment* or outdoor or natur* or natur* environment* or natur* connect* or natur* experience* or natur* therap* or ecotheap* or ecopsychosocial* or life style* or green* or out?door play* or Park* or greenspace* or green space* or ocean* or bluespace* or blue space* or surf* or sail* or voyage* or forest* or forest school* or garden* or golf* or biodiverse* or Horticultur* or leisure* or wild* or adventure* or Canine therap* or Equine therap* or Hippotherap* or Horse rid* or Adventur* therap* or wilderness* or camp* or summer camp* or out?patient* or communit* or experiential?learning) not (pharmacological* or pharmaceutical*)).mp. [mp=abstract, title, original title, heading words, cabicodes words]	1339777
3	Methods/ or Intervention*.mp. or therap*.mp. or teach*.mp. or program*.mp. or package*.mp. or support*.mp. or strateg*.mp. or service*.mp. or learning*.mp. [mp=abstract, title, original title, heading words, cabicodes words]	1812848
4	2 and 3	561632
5	1 and 4	379704
6	Child/ or Child*.mp. or youth*.mp. or adolesc*.mp. or teen*.mp. [mp=abstract, title, original title, heading words, cabicodes words]	478910
7	Autism Spectrum Disorder/ or Autistic Disorder/ or Asperger Syndrome/	25
8	((autis* or high function* autis* or Asperger* or pervasive development disorder* or neurodevelopmental disorder) not (ADHD or attention deficit hyperactivity disorder)).mp. [mp=abstract, title, original title, heading words, cabicodes words]	3727
9	7 or 8	3728
10	6 and 9	2480
11	5 and 10	392
12	((Randomized controlled trial or case control study or experimental* or quasi?experimental* or controlled clinical trial or randomized clinical trials or randomi* or random allocation or double blind* method or single blind* method) not (protocol* or guideline* or procedure* or polic* or review or meta analysis or systematic review)).mp. [mp=abstract, title, original title, heading words, cabicodes words]	296444
13	11 and 12	39

© 2023 Fan MSN et al. *JAMA Network Open.*

eTable 9. Medline Search Results

MEDLINE via Ovid**,** Ovid MEDLINE(R) <1946 to April 25, 2023>

1	(Health?related outcome* or Communication* or cooperation or decision making or equity or health* or well?being or independent* or flexib* or interpersonal or life?skill* or personal development or pro?social* or pro?social behavior* or resilien* or psycho?social or self?concept or self?confiden* or self?esteem or soci?emotion* or team?work or Quality of life or self?efficacy or psycho*).mp. [mp=title, book title, abstract, original title, name of substance word, subject heading word, floating sub-heading word, keyword heading word, organism supplementary concept word, protocol supplementary concept word, rare disease supplementary concept word, unique identifier, synonyms, population supplementary concept word, anatomy supplementary concept word]	6871789
2	((Nature?based* or recreation* or environment* or outdoor or natur* or natur* environment* or natur* connect* or natur* experience* or natur* therap* or ecotheap* or ecopsychosocial* or life style* or green* or out?door play* or Park* or greenspace* or green space* or ocean* or bluespace* or blue space* or surf* or sail* or voyage* or forest* or forest school* or garden* or golf* or biodiverse* or Horticultur* or leisure* or wild* or adventure* or Canine therap* or Equine therap* or Hippotherap* or Horse rid* or Adventur* therap* or wilderness* or camp* or summer camp* or out?patient* or communit* or experiential?learning) not (pharmacological* or pharmaceutical*)).mp. [mp=title, book title, abstract, original title, name of substance word, subject heading word, floating sub-heading word, keyword heading word, organism supplementary concept word, protocol supplementary concept word, rare disease supplementary concept word, unique identifier, synonyms, population supplementary concept word, anatomy supplementary concept word]	4893100
3	Methods/ or Intervention*.mp. or therap*.mp. or teach*.mp. or program*.mp. or package*.mp. or support*.mp. or strateg*.mp. or service*.mp. or learning*.mp. [mp=title, book title, abstract, original title, name of substance word, subject heading word, floating sub-heading word, keyword heading word, organism supplementary concept word, protocol supplementary concept word, rare disease supplementary concept word, unique identifier, synonyms, population supplementary concept word, anatomy supplementary concept word]	17248338
4	2 and 3	3473162
5	1 and 4	1174760
6	Child/ or Child*.mp. or youth*.mp. or adolesc*.mp. or teen*.mp. [mp=title, book title, abstract, original title, name of substance word, subject heading word, floating sub-heading word, keyword heading word, organism supplementary concept word, protocol supplementary concept word, rare disease supplementary concept word, unique identifier, synonyms, population supplementary concept word, anatomy supplementary concept word]	3724032
7	Autism Spectrum Disorder/ or Autistic Disorder/ or Asperger Syndrome/	41695
8	((autis* or high function* autis* or Asperger* or pervasive development disorder* or neurodevelopmental disorder) not (ADHD or attention deficit hyperactivity disorder)).mp. [mp=title, book title, abstract, original title, name of substance word, subject heading word, floating sub-heading word, keyword heading word, organism supplementary concept word, protocol supplementary concept word, rare disease supplementary concept word, unique identifier, synonyms, population supplementary concept word, anatomy supplementary concept word]	56546
9	7 or 8	58714
10	6 and 9	39206
11	5 and 10	4140

© 2023 Fan MSN et al. *JAMA Network Open.*

12	((Randomized controlled trial or case control study or experimental* or quasi?experimental* or controlled clinical trial or randomized clinical trials or randomi* or random allocation or double blind* method or single blind* method) not (protocol* or guideline* or procedure* or polic* or review or meta analysis or systematic review)).mp. [mp=title, book title, abstract, original title, name of substance word, subject heading word, floating sub-heading word, keyword heading word, organism supplementary concept word, protocol supplementary concept word, rare disease supplementary concept word, unique identifier, synonyms, population supplementary concept word, anatomy supplementary concept word]	1923733
13	11 and 12	287

© 2023 Fan MSN et al. *JAMA Network Open*.

eTable 10. PsycInfo Search Results

APA PsycInfo via Ovid**,** APA PsycInfo <1806 to April Week 3 2023>

1	(Health?related outcome* or Communication* or cooperation or decision making or equity or health* or well?being or independent* or flexib* or interpersonal or life?skill* or personal development or pro?social* or pro?social behavior* or resilien* or psycho?social or self?concept or self?confiden* or self?esteem or soci?emotion* or team?work or Quality of life or self?efficacy or psycho*).mp. [mp=title, abstract, heading word, table of contents, key concepts, original title, tests & measures, mesh word]	2883907
2	((Nature?based* or recreation* or environment* or outdoor or natur* or natur* environment* or natur* connect* or natur* experience* or natur* therap* or ecotheap* or ecopsychosocial* or life style* or green* or out?door play* or Park* or greenspace* or green space* or ocean* or bluespace* or blue space* or surf* or sail* or voyage* or forest* or forest school* or garden* or golf* or biodiverse* or Horticultur* or leisure* or wild* or adventure* or Canine therap* or Equine therap* or Hippotherap* or Horse rid* or Adventur* therap* or wilderness* or camp* or summer camp* or out?patient* or communit* or experiential?learning) not (pharmacological* or pharmaceutical*)).mp. [mp=title, abstract, heading word, table of contents, key concepts, original title, tests & measures, mesh word]	1293860
3	Methods/ or Intervention*.mp. or therap*.mp. or teach*.mp. or program*.mp. or package*.mp. or support*.mp. or strateg*.mp. or service*.mp. or learning*.mp. [mp=title, abstract, heading word, table of contents, key concepts, original title, tests & measures, mesh word]	2694011
4	2 and 3	747290
5	1 and 4	476796
6	Child/ or Child*.mp. or youth*.mp. or adolesc*.mp. or teen*.mp. [mp=title, abstract, heading word, table of contents, key concepts, original title, tests & measures, mesh word]	1215273
7	Autism Spectrum Disorder/ or Autistic Disorder/ or Asperger Syndrome/	53752
8	((autis* or high function* autis* or Asperger* or pervasive development disorder* or neurodevelopmental disorder) not (ADHD or attention deficit hyperactivity disorder)).mp. [mp=title, abstract, heading word, table of contents, key concepts, original title, tests & measures, mesh word]	65319
9	7 or 8	67871
10	6 and 9	47194
11	5 and 10	5162
12	((Randomized controlled trial or case control study or experimental* or quasi?experimental* or controlled clinical trial or randomized clinical trials or randomi* or random allocation or double blind* method or single blind* method) not (protocol* or guideline* or procedure* or polic* or review or meta analysis or systematic review)).mp. [mp=title, abstract, heading word, table of contents, key concepts, original title, tests & measures, mesh word]	303859
13	11 and 12	318

© 2023 Fan MSN et al. *JAMA Network Open.*

eTable 11. SPORTDiscus Search Results

EBSCOhost

Thursday, April 27, 2023 4:27:18 AM

#	Query	Limiters/Expanders	Last Run Via	Results
S9	S5 AND S8	Expanders - Apply equivalent subjects Search modes - Boolean/Phrase	Interface - EBSCOhost Research Databases Search Screen - Advanced Search Database - SPORTDiscus with Full Text	282
S8	S6 AND S7	Expanders - Apply equivalent subjects Search modes - Boolean/Phrase	Interface - EBSCOhost Research Databases Search Screen - Advanced Search Database - SPORTDiscus with Full Text	1,460
S7	(Autism Spectrum Disorder/ OR Autistic Disorder/ OR Asperger Syndrome/) OR ((autis* OR high function* autis* OR Asperger* OR pervasive development disorder* OR neurodevelopmental disorder)) NOT (ADHD OR attention deficit hyperactivity disorder)	Expanders - Apply equivalent subjects Search modes - Boolean/Phrase	Interface - EBSCOhost Research Databases Search Screen - Advanced Search Database - SPORTDiscus with Full Text	2,191
S6	Child/ OR (Child* OR youth* OR adolesc* OR teen*)	Expanders - Apply equivalent subjects Search modes - Boolean/Phrase	Interface - EBSCOhost Research Databases Search Screen - Advanced Search Database - SPORTDiscus with Full Text	193,593
S5	S1 AND S4	Expanders - Apply equivalent subjects Search modes - Boolean/Phrase	Interface - EBSCOhost Research Databases Search Screen - Advanced Search Database - SPORTDiscus with Full Text	117,453
S4	S2 AND S3	Expanders - Apply equivalent subjects Search modes - Boolean/Phrase	Interface - EBSCOhost Research Databases Search Screen - Advanced Search Database - SPORTDiscus with Full Text	214,638
S3	Methods/ OR (Intervention* OR therap* OR teach* OR program* OR package* OR support* OR strateg* OR service* OR learning*)	Expanders - Apply equivalent subjects Search modes - Boolean/Phrase	Interface - EBSCOhost Research Databases Search Screen - Advanced Search Database - SPORTDiscus with Full Text	776,941
S2	((Nature?based* OR recreation* OR environment* OR outdoor OR natur* OR natur* environment* OR natur* connect* OR natur* experience* OR natur* therap* OR ecotheap* OR ecopsychosocial* OR life style* OR green* OR out?door play* OR Park* OR greenspace* OR green space* OR ocean* OR bluespace* OR blue space* OR surf* OR sail* OR voyage* OR forest* OR forest school* OR garden* OR golf* OR biodiverse* OR Horticultur* OR leisure* OR wild* OR adventure* OR Canine therap* OR Equine therap* OR Hippotherap* OR Horse rid* OR Adventur* therap* OR wilderness* OR camp* OR summer camp* OR out?patient* OR communit* OR experiential?learning)) NOT ((pharmacological* OR pharmaceutical*))	Expanders - Apply equivalent subjects Search modes - Boolean/Phrase	Interface - EBSCOhost Research Databases Search Screen - Advanced Search Database - SPORTDiscus with Full Text	616,674
S1	Health?related outcome* OR Communication* OR cooperation OR decision making OR equity OR health* OR well?being OR independent* OR flexib* OR interpersonal OR life?skill* OR personal development OR pro?social* OR pro?social behavior* OR resilien* OR psycho?social OR self?concept OR self?confiden* OR self?esteem OR soci?emotion* OR team?work OR Quality of life OR self?efficacy OR psycho*	Expanders - Apply equivalent subjects Search modes - Boolean/Phrase	Interface - EBSCOhost Research Databases Search Screen - Advanced Search Database - SPORTDiscus with Full Text	651,260

© 2023 Fan MSN et al. *JAMA Network Open*.

eTable 12. Web of Science Search Results

https://www.webofscience.com/wos/woscc/summary/f2a4b519-daa2-47b2-9a8a-1aedad5c7911-85a83394/relevance/1

**Health related outcome*** (All Fields) and **Nature based* OR recreation* OR environment* OR outdoor OR natur* OR natur* therap* OR ecotheap* OR ecopsychosocial* OR life style* OR green* OR out?door play* OR Park* OR greenspace* OR green space* OR ocean* OR bluespace* OR blue space* OR surf* OR sail* OR voyage* OR forest* OR forest school* OR garden* OR golf* OR Horticultur* OR leisure* OR wild* OR adventure* OR Canine therap* OR Equine therap* OR Hippotherap* OR Horse rid* OR Adventur* therap* OR wilderness* OR camp* OR summer camp*** (All Fields) and **Intervention* OR therap* OR teach* OR program* OR package* OR support* OR strateg* OR service* OR learning*** (All Fields) and **Child* OR youth* OR adolesc* OR teen*** (All Fields) and **autis* OR high function* autis* OR Asperger* OR pervasive development disorder* OR neurodevelopmental disorder** (All Fields) and **Randomized controlled trial OR case control study OR experimental* OR quasi?experimental* OR controlled clinical trial OR randomized clinical trials OR randomi* OR random allocation OR double blind* method OR single blind* method** (All Fields)	152

© 2023 Fan MSN et al. *JAMA Network Open*.

eTable 13. Summary Description of the Characteristics of included studies

Authors	Country	Study design	Sample size	Ages (Mean (SD), Range)	% of female	Category under rehabilitation	Element(s) of nature	Components in nature interaction(s)	Attrition rate
Anderson and Meints (2016)	UK	PPS	15	10 (3.8) range: 5-16	26.7	Recreation therapy	Equine-assisted activities in a horse-riding center with outdoor open spaces	(1) (2)	nd
Ajzenman et al. (2013)	USA	PPS	7	8.4 (2.5) Range: 5-12	42.9	Recreation therapy	Hippotherapy in the equestrian center with pastures	(1) (2)	14.3%
Bass et al. (2009)	USA	RCT	N:34 I: 19 C: 15	1: 6.95 (1.67) Range: 5-10 C: 7.73 (1.65) Range: 4-10	14.7	Recreation therapy	Therapeutic horseback riding in an equestrian training center with outdoor open spaces	(1) (2)	26.5%
Borgi et al. (2016)	Italy	RCT	N:28 I: 15 C: 13	8.6 (1.7) Range: 6-12	0	Recreation therapy	Equine-assisted therapy program in a horse-riding center with outdoor open spaces	(1) (2)	0
Cavanaugh and Rademacher (2014)	USA	PPS	11	SD1: 13.2 (2.2) Range: 10-16 SD2: 12.6 (1.8) Range: 11-15	18.2	Recreation therapy	Surf camp on local lake and ocean beach	(1) (2)	nd
Coman et al. (2018)	USA	RCT	N: 50 I: 25 C: 25	8.7 (1.6) Range: 7-12	16.0	Recreation therapy	Equine-assisted activities in an equestrian training center with outdoor open spaces	(1) (2)	0
Gabriels et al. (2015)	USA	RCT	N:116 I: 58 C: 58	10.2 (3.0) Range: 6-16	12.9	Recreation therapy	Therapeutic horseback riding in a horse riding center with outdoor open spaces	(1) (2)	23.3%
Garcia-Gomez et al. (2014)	Spain	QED-C	N: 16 I: 8 C: 8	Range: 7-14	18.8	Recreation therapy	Adapted therapeutic horseback riding in an equestrian training center with outdoor open spaces	(1) (2)	nd

© 2023 Fan MSN et al. *JAMA Network Open.*

Harris and Williams (2017)	UK	QED-C	N: 26<br>I: 12<br>C: 14	7.5 (10.57)<br>Range: 6-9	15.4	Recreation therapy	Horse-riding intervention in a horse-riding center with outdoor open spaces	(1) (2)	7.7%
Hemati et al. (2013)	Iran	PPS	6	8.5 (2.35)<br>Range: 6-12	83.3	Recreation therapy	Therapeutic horseback riding with outdoor open spaces	(1) (2)	0
Ibrahim and Cronin (2020)	USA	PPS	40	Range: 5-21	10.0	Recreation therapy	Summer camp program with nature-based activities, namely hiking, fishing, horseback riding, and camping.	(1) (2)	nd
Kern and Aldridge (2006)	Canada	PPS	4	Range: 3-5	0	Music therapy	Outdoor play with embedded music therapy in an outdoor playground with a wooden playhouse, green playfield, flowers and garden beds, and a music hut.	(1) (2)	nd
Kern et al. (2011)	USA	PPS	41	7.8 (2.9)<br>Range: 3-12	25.0	Recreation therapy	Equine-assisted activities in an equestrian center with outdoor open spaces	(1) (2)	41.5%
Lanning et al. (2014)	USA	QED-C	N:25<br>I: 10<br>C: 8	I: 7.5 (3.2)<br>Range: 4-15<br>C: 9.8 (2.82)<br>Range: 5-14	16.0	Recreation therapy	Equine-assisted activities in equestrian training centers with outdoor open spaces	(1) (2)	0
Parmar and Patel (2022)	India	PPS	30	8.6 (2.4)<br>Range: 5-14	53.3	Art therapy	Art therapy in an outdoor garden	(1) (2)	nd
Pan et al. (2019)	USA	RCT	N: 16<br>I: 8<br>C: 8	I: 11.88 (2.45)<br>C: 9.8 (2.82)<br>Range: 6-16	18.9	Recreation therapy	Therapeutic horseback riding in a horse-riding center with outdoor open spaces	(1) (2)	12.5%
Peters et al. (2022)	USA	RCT	N: 24<br>I: 12<br>C: 9	I: 8.68 (2.09)<br>C: 9.45 (1.62)<br>Range: 6-13	20.8	Recreation therapy	Occupational therapy in an equine environment (Garden)	(1) (2)	28.6%
Ramshini et al. (2018)	Iran	QED-C	N: 14<br>I: 7<br>C:7	Range: 3-7	14.3	Nature therapy	Nature therapy in outdoor wood (Sitting and lying on the lawn, collecting stones, covering body with soil and mud, collecting wood to start fire, walking on dry tree leaves, collecting vegetables, and interacting with animals).	(1) (2) (3)	nd
Scartazza et al. (2020)	Italy	PPS	8	17.8 (3.2)<br>Range: 15-23	0	Horticultural therapy	Horticultural therapy in a healing garden (aromatic spiral, raised beds, flowerbeds, kitchen garden/orchard, olive, aromatic hedges, and fruit trees	(1) (2) (3)	nd
Shanok et al. (2019)	USA	PPS	46	11.46 (6.21)<br>Range: 6-24	19.6	Recreation therapy	Golfing program in a golf course with a large field of grass	(1) (2)	nd
Souza-Santos et al. (2018)	Brazil	Crossover RCT	N:45<br>I_1:15<br>I_2: 15<br>I_3: 15	I_1: 7 (1.09)<br>I_2: 7 (1.09)<br>I_3: 7 (1.09)<br>Range: 5-12	20.0	Recreation therapy	Equine-assisted therapy in outdoor space	(1) (2)	0

© 2023 Fan MSN et al. *JAMA Network Open*.

Ward et al. (2013)	USA	PPS	21	8.1 (n.d.)<br>Range: nd	28.6	Recreation therapy	Therapeutic horseback riding in a therapeutic riding center with outdoor open spaces	(1) (2)	nd
Zachor et al. (2016)	Israel	QED-C	N: 51<br>I: 30<br>C: 21	5.33 (0.92)<br>Range: 3-7	21.6	Recreation therapy	Outdoor adventure activities in the community park.	(1) (2)	nd
Zhao et al. (2021)	China	QED-C	N: 61<br>I: 31<br>C: 30	7.1 (1.42)<br>Range: 6-12	27.9	Recreation therapy	Therapeutic horseback riding in an equestrian training center with outdoor open spaces	(1) (2)	19.7%

[1] RCT: Randomized controlled trial; QED-C: Quasi-experimental design with control group; PPS: Pre-post study.
[2] N: Total number of the participant; I: Intervention group (Experimental group); C: control group
[3] (1) - place-based, (2) - feature active bodily engagement, (3) recognize nature-human kinship
[4] nd: No data; [5] SD: school district

© 2023 Fan MSN et al. *JAMA Network Open*.

eTable 14. Summary Description of the Intervention of Included Studies

Authors	Theoretical framework	Intervention								Control
		Content	Delivery mode	Teaching and learning modality	Program duration (week)	Session frequency (per week)	Session length (minutes)	Intervenors	Format	
Anderson and Meints (2016)	nd	5 sessions<br>(1) Therapeutic riding<br>(2) Horsemanship<br>(3) Stable management	Face-to-face	· Experiential learning<br>· Therapeutic intervention<br>· Natural horsemanship	5	1	180	· Instructors with BHS and RDA qualifications<br>· Experienced volunteers	Group-based	No
Ajzenman et al. (2013)	nd	12 sessions<br>Hippotherapy based on<br>(1) Motor control<br>(2) Functional communication<br>(3) Cognition<br>(4) Social skills<br>(5) Interactive play	Face-to-face	· Experiential learning<br>· Therapeutic intervention<br>· Natural horsemanship	12	1	45	State-licensed occupational therapists or certified occupational therapy assistants working with a PATH-certified instructor	Group-based	No
Bass et al. (2009)	nd	12 sessions<br>(1) Therapeutic riding<br>(2) Mounting/dismounting<br>(3) Exercises<br>(3) Mounted games<br>(4) Horsemanship activities.	Face-to-face	· Experiential learning<br>· Therapeutic intervention<br>· Natural horsemanship	12	1	60	Trained Good Hope Equestrian Training Center (GHETC) instructors and volunteers	Group-based	Waitlist control
Borgi et al. (2016)	nd	24 sessions<br>(1) Therapeutic riding<br>(2) Structured activities working on the ground<br>(3) Structured activities working with horses	Face-to-face	· Experiential learning<br>· Therapeutic intervention<br>· Natural horsemanship	24	1	60-70	FISE-certified riding instructors	Group-based	Waitlist control
Cavanaugh and Rademacher (2014)	nd	Two-day Surf camp with the following components<br>(1) Family socials<br>(2) Social skills & group activities	Face-to-face	· Experiential learning	nd	nd	nd	Volunteers included occupational, physical, and speech therapists, teachers, and university students	Group-based	No
Coman et al. (2018)	nd	12 sessions<br>(1) Therapeutic riding<br>(2) Mounting/dismounting,<br>(3) Exercises	Face-to-face	· Experiential learning<br>· Therapeutic intervention	12	1	60	A certified PATH International advanced therapeutic riding instructor and volunteers	Group-based	Waitlist control

© 2023 Fan MSN et al. *JAMA Network Open.*

		(4) Mounted games<br>(5) Horsemanship activities.								
Gabriels et al. (2015)	nd	<u>10 sessions</u><br>(1) Therapeutic riding<br>(2) Horsemanship	Face-to-face	· Experiential learning<br>· Therapeutic intervention<br>· Natural horsemanship	10	1	45	A certified PATH International advanced therapeutic riding instructor and volunteers	Group-based	Placebo (Barn activities)
Garcia-Gomez et al. (2014)	nd	<u>24 sessions</u><br>(1) Therapeutic riding<br>(2) Preparing the equipment and the horse<br>(3) Mounting/ dismounting<br>(4) Horsemanship	Face-to-face	· Experiential learning<br>· Therapeutic intervention<br>· Natural horsemanship	12	2	45	Instructors with PATH International training	Group-based	Usual care
Harris and Williams (2017)	nd	<u>5-7 sessions</u><br>(1) Therapeutic riding<br>(2) Preparation and mounting<br>(3) Stretching exercise<br>(4) Thanking instructors and horses	Face-to-face	· Experiential learning<br>· Therapeutic intervention<br>· Natural horsemanship	5-7	1	45	Instructors who are accredited by the BHS and sider walkers (school teaching staff or volunteers)	Group-based	Waitlist
Hemati et al. (2013)	nd	<u>8 sessions</u><br>(1) Familiarity stage<br>(2) Practices<br>(3) Therapeutic riding skills<br>(4) End of riding stage	Face-to-face	· Experiential learning<br>· Therapeutic intervention<br>· Natural horsemanship	4	2	45	Two experienced trainers with assistance from parents and teachers	Group-based	No
Ibrahim and Cronin (2020)	nd	Summer camp program "New Amigo" includes arts, and crafts, sports, games, swimming, horseback riding, music, archery, kayaking, and wall climbing.	Face-to-face	· Experiential learning	1	nd	33	Camp staff composed of 55 volunteer counselors, including university students, community professionals, and volunteers	Group-based	No
Kern and Aldridge (2006)	nd	Outdoor play with embedded music therapy. Having morning playground time with or without adaptation of the playground, with teacher-mediated intervention or peer-mediated intervention, in an outdoor music hut	Face-to-face	· Experiential learning	nd	nd	nd	School-led teachers and assistant teachers	Group-based	No
Kern et al. (2011)	nd	<u>24 sessions</u><br>(1) Therapeutic riding<br>(2) Horse management, incorporating responsibility and ownership of an assigned horses	Face-to-face	· Experiential learning<br>· Therapeutic intervention<br>· Natural horsemanship	24	1	60	Horse riding instructors and parents as side walkers	Group-based	No

© 2023 Fan MSN et al. *JAMA Network Open.*

		(3) Leading and brushing the horses<br>(4) Skills in putting on the bridle and saddle								
Lanning et al. (2014)	nd	12 sessions<br>(1) Basic safety lessons (meeting the horse, respecting the horse, putting on the helmet)<br>(2) Grooming lessons<br>(3) Riding activities	Face-to-face	· Experiential learning<br>· Natural horsemanship	12	1	60	Certified riding instructors and volunteers	Group-based	Placebo (Structured social circle)
Parmar and Patel (2022)	nd	<u>36 sessions</u><br>Outdoor art therapy<br>(1) Drawing therapy<br>(2) Clay therapy<br>(3) Drama therapy<br>(4) Dance therapy<br>(5) Strengthening exercise	Face-to-face	· Experiential learning	6	6	nd	nd	Group-based	No
Pan et al. (2019)	nd	<u>10 sessions</u><br>(1) Therapeutic riding skills (mounting, halting, steering, turning, and trotting)<br>(2) Horsemanship skills (how to lead and care for horses)<br>(3) Following routines (putting on riding helmet, waiting on the bench, mounting horse, riding activities, dismounting horse, grooming horse, putt away equipment)	Face-to-face	· Experiential learning<br>· Therapeutic intervention<br>· Natural horsemanship	10	1	45	A certified PATH International advanced therapeutic riding instructor and volunteers	Group-based	Placebo (Barn activities)
Peters et al. (2022)	nd	9-10 sessions<br>OTee Horsplay<br>(1) Greetings<br>(2) Activities with horses<br>(3) Saying goodbyes<br>(4) Caregiver debriefing	Face-to-face	· Experiential learning<br>· Therapeutic intervention<br>· Natural horsemanship	9-10	1	60	Occupational therapist who completed Hippotherapy Treatment Principles Part 1 and 2 training and PATH International registered instructor	Individual	Waitlist control
Ramshini et al. (2018)	nd	10 sessions<br>Family-centered nature therapy<br>(1) Horticultural therapy activities<br>(2) Establishing relationships with animals in nature<br>(3) Physical activities (with focuses on the five senses in nature.	Face-to-face	· Experiential learning<br>· Environment education	10	1	90	Specialists from health centers and staff of the Savan Nature School.	Individual family	Usual care

© 2023 Fan MSN et al. *JAMA Network Open*.

Scartazza et al. (2020)	nd	96 sessions<br>Conservation programs of crop landraces, through the interactions among the participants, health care professionals, and local and scientific community.	Face-to-face	· Experiential learning<br>· Environment education	96	1	240	Healthcare-educational professionals, researchers, elderly farmers, and volunteers	Group-based	No
Shanok et al. (2019)	nd	12 sessions<br>Ernie Els #GameON Autism ™ Golf Program delivers golf instruction (distance control, getting the ball in the air, aiming at the target, and taking practice swings) incorporated with targeted autism learning objectives focusing on<br>(1) Communication skills<br>(2) Regulatory skills<br>(3) Motor skills<br>(4) Social skills using visual cues.	Face-to-face	· Experiential learning	6	2	45	Professional golf instructors and volunteers	Group-based	No
Souza-Santos et al. (2018)	nd	24 sessions<br>(1) Equine-assisted therapy consisted of horse approach, touch stimulation, ride, and course with varied riding.<br>(2) Dance program "TALT" consisted of warm-up (body conditioning), assembly of choreographies (body coordination, memory, perception, and rhythm), and relaxation.<br>(3) Dance with equine-assisted therapy	Face-to-face	· Experiential learning<br>· Therapeutic intervention<br>· Natural horsemanship	12	2	60	Qualified therapist and trained dance master	Group-based	Crossover RCT
Ward et al. (2013)	nd	Therapeutic horseback riding comprises Orientation (from early sensory activities to touching and grooming the horse), mounting and quiet riding, riding skills, and closure.	Face-to-face	· Experiential learning<br>· Therapeutic intervention	18	nd	nd	A PATH registered level instructor, a trained horse leader, and two trained side walkers and volunteers	Group-based	No
Zachor et al. (2016)	nd	13 sessions<br>The outdoor adventure program (1) Opening songs<br>(2) Using the rope devices	Face-to-face	· Experiential learning	13	1	30	The outdoor adventure program staff, including a senior guide and two field instructors.	Group-based	Usual care

© 2023 Fan MSN et al. *JAMA Network Open.*

		(3) Outdoor adventure program with the use of a two-way climbing rope ladder, rope elevator, rope bridge, and a hammock and rope swing								
Zhao et al. (2021)	nd	32 sessions<br>Therapeutic riding consisted of structured activities and exercises that addressed social skills, communication skills, and horsemanship skills.	Face-to-face	· Experiential learning<br>· Therapeutic intervention<br>· Natural horsemanship	16	2	60	Certified therapeutic riding instructors, trained volunteers	Group-based	Usual care

© 2023 Fan MSN et al. *JAMA Network Open*.

eFigure 1. Revised Cochrane Risk-of-Bias tool for randomized trials (RoB 2) Summary and Author Judgments of Low, Some concerns, and High Risk of Bias Across All Included RCTs studies (N = 7)

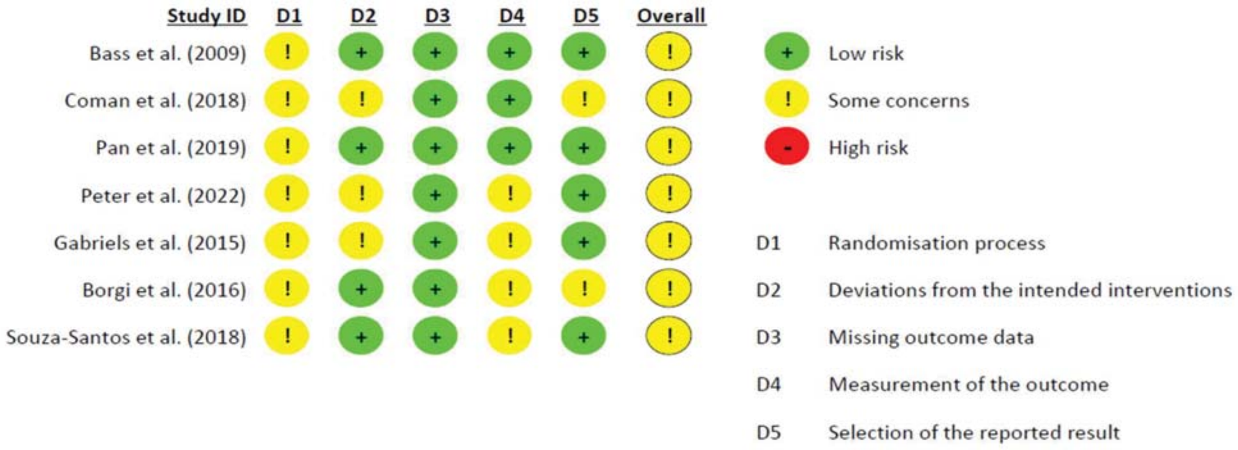

© 2023 Fan MSN et al. *JAMA Network Open.*

eFigure 2. Cochrane Risk of Bias in Non-randomized Studies – of Intervention (ROBINS-I) Summary and Author Judgments of Low, Moderate, serious, and Critical Risk of Bias Across All Included Non-RCTs Studies (N = 17)

Study	Bias due to confounding	Bias in selection of participants into the study	Bias in classification of interventions	Bias due to deviations from intended interventions	Bias due to missing data	Bias in measurement of outcomes	Bias in selection of the reported result	Overall Bias
Ajzenman et al. (2013)	Low	Moderate	Low	Moderate	Moderate	Serious	Serious	Critical
Anderson and Meints (2016)	Low	Serious	Low	Moderate	Moderate	Serious	Serious	Critical
Cavanaugh and Rademacher (2014)	Moderate	Moderate	Low	Moderate	Moderate	Serious	Serious	Critical
Garcia-Gomez et al. (2014)	Low	Low	Low	Low	Low	Moderate	Moderate	Low
Harris and Williams (2017)	Low	Moderate	Low	Low	Low	Moderate	Moderate	Moderate
Hemati et al. (2013)	Moderate	Moderate	Low	Moderate	Moderate	Serious	Serious	Critical
Ibrahim and Cronin (2020)	Moderate	Moderate	Moderate	Moderate	Moderate	Serious	Serious	Critical
Kern et al. (2011)	Low	Low	Low	Moderate	Moderate	Serious	Serious	Critical
Kern and Aldridge (2006)	Moderate	Moderate	Low	Moderate	Moderate	Serious	Serious	Critical
Lanning et al. (2014)	Low	Moderate	Low	Low	Low	Moderate	Moderate	Moderate
Parmar and Patel (2022)	Moderate	Moderate	Moderate	Moderate	Moderate	Serious	Serious	Critical
Ramshini et al. (2018)	Moderate	Low	Low	Low	Low	Moderate	Low	Moderate
Scartazza et al. (2020)	Moderate	Moderate	Moderate	Moderate	Moderate	Serious	Serious	Critical
Shanok et al. (2019)	Low	Moderate	Low	Moderate	Moderate	Serious	Serious	Critical
Ward et al. (2013)	Low	Low	Low	Moderate	Moderate	Serious	Serious	Critical
Zachor et al. (2016)	Low	Low	Low	Low	Moderate	Moderate	Low	Low
Zhao et al. (2021)	Low	Low	Low	Low	Low	Low	Low	Low

© 2023 Fan MSN et al. *JAMA Network Open.*

eTable 15. Summary Description of the Outcomes and Measurements of Included Studies

Authors	Measurements	Measured domains	Follow-up duration	Key findings
Anderson and Meints (2016)	(1) Autism spectrum quotient (ASQ-child and ASU-adolescents); (2) The Vineland Adaptive Behavior Scale; (3) the empathizing and systemizing quotient<br><br>Rated by parent (1, 3)<br>Completed with the parent by the researcher	Social skills, communication skills, imagination, attention to detail, attention switching/ tolerance of change, communication, daily living skills, socialization, motor skills, and maladaptive behavior index.	No	(1) ASD traits ↓ [F (1,10) =11.195, P=0.007, $\eta^2\rho$=0.528]<br>(2) The VABS adaptive score →; communication →; socialization →; Maladaptive behavior trait ↓ [F (1,11) =5.65, P=0.037, $\eta^2\rho$=0.339]]<br>(3) Empathizing ↑ [F (1,11) =5.19, P=0.04, $\eta^2\rho$=0.320]; systematizing →
Ajzenman et al. (2013)	(1) Force Plates and Video Motion Capture ;(2) The Vineland Adaptive Behavior Scale (VABS); (2) Child Activity Card Sort (CACS)<br><br>Rated by parent (1-2)	(1) Motor control and postural stability, (2) adaptive behavior (adaptive composite score, communication, daily living skills, socialization, motor skills; and (3) participation (self-care, community mobility, high-demand leisure, low-demand leisure, social interaction, domestic, education)	No	(1) Postural stability ↑<br>(2) Overall adaptive behavior composite score ↑ (d=0.393, p=0.027); communication ↑ (d=0.473, p=0.042); daily living skills →; socialization ↑ (d=0.364, p=0.027); motor skills →<br>(3) Self-care ↑ (d=0.624, P-0.027), community mobility→, high-demand leisure→, low-demand leisure ↑ (d=0.889, P=0.042), social interaction ↑ (d=0.911, p=0.027), domestic, education→.
Bass et al. (2009)	(1) Social Responsiveness Scale (SRS); (2) Sensory Profile (SP)<br><br>Rated by parent (1,2)	(1) social awareness, social cognition, social communication, social motivation, and autism mannerisms; (2) fine motor/ perception, sensory seeking, attention and distractibility, sensory sensitivity, and sedentary.	No	(1) SRS overall score ↑ [F (1,20) =4.92, P=0.038, η^2=0.20]; social motivation ↑ [F (1,25) =4.80, P=0.038, η^2=61; social cognition →; social awareness →<br>(2) Sensory Profile overall score ↑ [F (1,31) =10.98, P=0.002, η^2=0.26]; fine motor/ perception →; sensory seeking ↑ [F (1,30) =17.09, P<0.01, η^2=0.40]; attention and distractibility ↑ [F (1,29) =19.17, P,0.01, η^2=0.40]; sensory sensitivity ↑ [F (1,31) =31.01, P,0.01, η^2=0.50]; sedentary ↑ [F (1,31) =18.59, P,0.01, η^2=0.375];
Borgi et al. (2016)	(1) The Vineland Adaptive Behavior Scale (VABS); (2) The Tower of London (TOL)<br><br>Semi-structured interview with parent/ legal guardian (1)<br>Rated by blinded evaluator (2)	(1) Communication, daily living skills, socialization, and motor skills.	No	Adaptive and executive functioning ↑ [F >9.68, all P<0.006].<br>(1) Communication →; daily living skills→; socialization ↑ [F (1,18) =5.30, P=0.034]; and motor skills. ↑ [F (1,10) =7.43, P=0.021],<br>(2) Total number of moves →, planning time ↑ [F (1,19) =5.58, P=0.026], execution time →, total problem-solving time →, number of correct solutions →, number of rule violation→

© 2023 Fan MSN et al. *JAMA Network Open.*

Cavanaugh and Rademacher (2014)	(1) Social Skills Improvement System (SSIS); (2) the 2nd edition of the Piers-Harries Children's Self Concept Scale (PH2); (3) the Parent Perceptions of the Surf Camp Curriculum (PPSCC); (4) the SURF Skills Checklist; (5) the SURF Camp Curriculum Activity Observation Checklist<br><br>Self-report (1)<br>Rate by parent (1)	(1) Communication, cooperation, assertion, responsibility, empathy, engagement, and self-control; (2) physical appearance and attributes, intellectual and school status, happiness and satisfaction, freedom from anxiety, behavioral adjustment, and popularity; (3) Parent perception of child's social competence, social skills, and self-concept; (4) social competence, social skills, and overall self-concept (stay-in-the-group; use-my-see-step; sound; expression eye-contact;	2 weeks	(1) Assertion↑ (P=0.01); responsibility ↑ (P=0.023); engagement ↑ (P=0.016) in both self-report and parent-report<br>(2) physical appearance and attributes→, intellectual and school status→, happiness and satisfaction→, freedom from anxiety→, behavioral adjustment→, and popularity→<br>(3) Paddle Relay activity ↑ (P=0.015) post-intervention but not significant in FU; Sandcastle Activity ↑?
Coman et al. (2018)	(1) Social Responsiveness Scale (SRS); (2) Sensory Profile (SP); (3) Sensory Profile School Companion (SPSC)<br><br>Rated by teacher and parent (1)<br>Rated by parent (2)<br>Rated by teachers (3)	(1) social cognition, social communication, social awareness, social motivation, autistic mannerisms; (2) sensation seeking, emotional reactive, low endurance/tone, oral sensory sensitivity, inattention/ distractibility, poor registration, sensory sensitivity, sedentary and fine motor/ perception; (3) registration, seeking, sensitivity, and avoiding	8 weeks	(1) SRS total score ↓ [F (1,48) =25.34, P<0.001, η²=0.35]; social cognition ↓ [F (1,48) =13.88, P=0.001, η²=0.22], social communication ↓ [F (1,48) =19.47, P<0.001, η²=0.29], social awareness →, social motivation ↓ [F (1,48) =24.40, P<0.001, η²=0.34], autistic mannerisms ↓ [F (1,48) =20.69, P<0.001, η²=0.30];<br>(2) SP total score ↑ [F (1,48) =21.09, P<0.001, η²=0.31; sensation seeking →, emotional reactive ↑ [F (1,48) =16.21, P<0.001, η²=0.25], low endurance/tone ↑ [F (1,48) =21.85, P<0.001, η²=0.31], oral sensory sensitivity→, inattention/ distractibility ↑ [F (1,48) =31.84, P<0.001, η²=0.40], poor registration →, sensory sensitivity →, sedentary ↑ [F (1,48) =19.57, P<0.001, η²=0.29]and fine motor/ perception→;<br>(3) registration ↑ [F (1,48) =19.08, P<0.001, η²=0.28], seeking ↑ [F (1,48) =6.84, P=0.012, η²=0.13], sensitivity↑ [F (1,48) =16.97, P<0.001, η²=0.26], and avoiding↑ [F (1,48) =21.97, P<0.001, η²=0.31].
Gabriels et al. (2015)	(1) Aberrant Behavior Checklist-Community (ABC-C); (2) the Peabody Picture Vocabulary Test, 4th ed. (PPVT-4); (3) Systematic Analysis of Language Transcripts (SALT); (4) The Bruininks-Oseretsky Test of Motor Proficiency, 2nd ed. (BOT-2) short form and two subscales of the Sensory Integration and Praxis Test (SIPT):	(1) Irritability, Lethargy/ social withdrawal, stereotypy, hyperactivity, and inappropriate speech; (2) Raw Score; (3) Number different words used, number words used; (4) Raw score; (5) Adaptive total score; (6) Social awareness, social cognition,	No	(1) Irritability ↑ (P=.02, ES=0.50); hyperactivity ↑ (P=.01, ES=0.53); (3) the use of different words ↑ (P=.01, ES=0.54); (4) BOT-2 AND SIPT→ (5) Adaptive score →; (6) Social cognition ↑ (P=.05, ES=0.41); Social communication ↑ (P=.01, ES=0.54)

© 2023 Fan MSN et al. *JAMA Network Open.*

	Praxis on Verbal Command and Postural Praxis; (5) The Vineland Adaptive Behavioral Scale, 2nd ed. (VABS-II); (6) The Social Responsive Scale (SRS)<br><br>Rated by caregiver (1, 6)<br>Rated by a speech therapist (2-3)<br>Rated by an occupational therapist (4)<br>Rated by a research assistant with caregiver	social communication, Autism Mannerisms, and social motivation		
Garcia-Gomez et al. (2014)	(1) Behavior Assessment System for Children (BASC-T) for teachers; (2) Quality-of-Life Model<br><br>Rated by teacher (1)<br>Rated by parent (2)	(1) Aggressiveness, hyperactivity, behavior problems, attention problems, learning problems, Atypicality, depression, anxiety, Withdrawal, somatization, externalized problems, internalizing problems, school problems, social skills, leadership, study skills, adaptive skills; (2) Emotional well-being, interpersonal relations, personal development, physical well-being, self-determination, social inclusion, familial well-being, total QoL	No	(1) Aggressiveness (p=0.039, d=0.220), hyperactivity→, behavior problems→, attention problems→, learning problems→, Atypicality→, depression→, anxiety→, Withdrawal→, somatization→, externalize problems→, internalize problems→, school problems→, social skills→, leadership →, study skills →, adaptive skills→<br>(2) Emotional well-being →, interpersonal relations (P=0.004, d=2.05), personal development →, physical well-being →, self-determination →, social inclusion (p=0.022, d=2.43), familial well-being→, total QoL→
Harris and Williams (2017)	(1) Childhood Autism Rating Scale, 2nd ed (CARS2), CARS standard version (CARS2-ST) for low-functioning participants and CARS2_HF) for 3 participants and (2) Aberrant Behavior Checklist-community edition (ABC-C); (3) Measurement of Pet Intervention Checklist (MOPI)<br><br>All rated by teachers.	(1) Severity of ASD core symptoms, (2) irritability, lethargy, stereotype, hyperactivity, and inappropriate speech; (3) Attention span, physical movement, verbal communication, and compliance.	No	(1) CARS2 ↓ [F (1,22) =7.219, P=0.013, ES=0.5]<br>(2) Hyperactivity ↓ [F (1,22) =8.084, P=0.009, ES=0.518]<br>(3) ¶
Hemati et al. (2013)	The social skills rating form, a subscale of the Triad Social Skill Assessment (TSSA)	Affective understanding/ perspective taking, initiating interactions, responding to interaction, and maintaining interaction.	No	Total score of social skills (t=3.75, P<0.05)<br>Affective understanding/ perspective taking ↑ (t=4.03, P=0.01), initiating interactions ↑ (t=3.78, P=0.01), responding to interaction →, and maintaining interaction ↑ (t=3.75, P=0.04).

© 2023 Fan MSN et al. *JAMA Network Open.*

Ibrahim and Cronin (2020)	Modified Autism Social Skill profile (ASSP) adapted from Bellini's (2008) Autism Social Skills Profile.<br><br>Rate by parents	Communication, reciprocity, social cognition, initiation, perspective-taking, and self-awareness.	8 weeks	Communication ↑ (P=0.019)<br>reciprocity↑ (P=0.037)<br>social cognition → (P=0.287)<br>initiation ↑ (P=0.027)<br>perspective taking and self-awareness → (P=0.158)<br>Combined domain ↑ (P<0.01)
Kern and Aldridge (2006)	Direct observation with a coding scheme<br><br>Rate by three independent observers	(1) Peer interactions on the playground (Initiate interaction, positive interaction); (2) Targeted children's interactions on the playground (Initiate interaction, positive interaction, stays in the music hut, play and engagement with material and equipment); (3) Teachers' task behaviors (Supported interactions, unsupported interaction)	No	¶
Kern et al. (2011)	(1) the Childhood Autism Rating Scale (CARS); (2) The Timberlawn Parent-child Interaction Scale; (3) Sensory Profile; (4) Quality of Life Enjoyment and Satisfaction Questionnaire: General Activities Subscale (QLES-Q); (5) Treatment Satisfaction Survey<br><br>Administered by a blinded research assistant (1, 2)<br>Rated by parent (3-5)	(2) Expressiveness, responsiveness, positive regard, negative regard, Mood and tone, and empathy	No	(1) Overall CARS score ↓ [F (2, 15)) =4.30, P<0.04)]<br>(2) all subdomains →<br>(3) all subscales →<br>(4) ↑ overall QoL [F (2, 14), P<0.02)]
Lanning et al. (2014)	(1) Pediatric Quality of Life 4.0 Generic Core Scales (PedsQL); (2) Child Health Questionnaire (CHQ)<br><br>Self-administered (1)<br>Rated by parent (1,2)	(1) Physical functioning, emotional function, social functioning, school functioning; (2) Physical functioning, Role/Social Emotion/ Behavioral, Role/Social limitations-physical, mental health, self-esteem, General health perception, parental impact-emotional, parent-impact-Time, and two aggregate summary score	No	(1) Physical functioning ↑ (△=13.6, SE=6.35, P=0.0415), emotional function ↑ (△=14.5, SE=6.68, P=0.0391), social functioning ↑ (△=22.5, SE=9.57, P=0.0263), Summary score psychosocial ↑ (△=13.0, SE=5.70, P=0.0308)
Pan et al. (2019)	(1) Systematic Analysis of Language Transcripts (SALT); (2) The Social Responsive Scale (SRS); (3) Aberrant Behavior Checklist-Community (ABC-C); (4) Saliva collection	(1) Improvement of the number of words or different words spoken; (2) social awareness, social cognition, social motivation, social	No	(1) of number wards or different words spoken →<br>(2) Social awareness (ES-1.74, P=0.01); Social communication (ES=-10.46, P=0.03);

© 2023 Fan MSN et al. *JAMA Network Open.*

	Conducted by a blinded speech therapist (1)<br>Rated by caregiver (2-3)<br>Collected by study personnel (4)	communication, and autistic mannerisms; (3) irritability, Lethargy/Social Withdrawal, Stereotype, Hyperactivity, and Inappropriate speech behavior		(3) Hyperactivity (ES=1.49, P=0.02); irritability subscale (ES=1.08, P=0.08)
Parmar and Patel (2022)	Childhood Autism Rating Scale (CARS)	nd	No	CARS score ↑ (P<0.01)
Peters et al. (2022)	(1) Goal Attainment Scaling (GAS); (2) Aberrant Behavior Checklist-community (ABC-C); (3) Social Responsive Scale 2nd ed. (SRS-2); (4) Pediatric Evaluation of Disability Evaluation of Disability Inventory Computer Adaptive Test, autism spectrum disorder version (PEDICAT-ASD)	(1) Primary goal, average of all goals; (2) irritability, Hyperactivity; (3) Social awareness, social cognition, social communication, social motivation, restricted and repetitive behavior; (4) Daily activities, Mobility, Social/cognitive, responsibility	No	(1) Primary goal ↑ via OT HORSPLAY (P<0.001) and control (P<0.001); Average of all goal ↑ via OT HORSPLAY (P<0.001) and control (P=.002);<br>(2) Social Motivation ↑ via OT HORSPLAY (P=.033) and control (P<0.001);<br>(4) →
Ramshini et al. (2018)	Child-Parent Relationship Scale (CPRS)<br><br>Rate by parents	Conflict; positive relationship; dependence; and parent-child relation	3 months	Conflict ↑; positive relationship ↑; dependence ↑. parent-child relation ↑
Scartazza et al. (2020)	(1) "undertaken a single task independently" (d2012) and "complex interpersonal interactions" (d720) in ICF-CY index; (2) Observational rating Scale of Basic Functions (SVFB)<br><br>Rated by the medical team, composed of experts in a neurological developmental disorder, healthcare, and educational professional	Initiative in expressing will; shared action or game; reaction to another's presence; and behavioral unpredictability.	No	d2012 in performance ↑ (*P*=.12)<br>d720 in performance ↑ (*P*=.28)<br>General behavior ↑ (i.e., indicating more high-functioning behavior) in intention (P=.018), interaction (P=.012), and regulation (P< .05).
Shanok et al. (2019)	Community perception survey<br><br>Rated by teachers and therapists	(1) Receptive communication skills; (2) expressive communication skills; (3) regulatory skills; (4) motor skills; (5) social skills; and (6) teachers' perception of the effectiveness of intervention	No	Receptive communication skills ↑ (P<0.001); expressive communication skills ↑ (p=0.004); regulatory skills ↑ (P<,0.001); motor skills ↑ (p<0.001); social skills ↑ (P=0.001)<br>Community perception survey ❡
Souza-Santos et al. (2018)	(1) Childhood Autism Rating Scale, (2) Functional Independence Measure, (3) WHO Disability Assessment schedule,	(2) self-care, transfers, locomotion, sphincter control, communication, and social cognition (memory, social interaction and problem-solving); (3) Cognition, mobility, self-care, coexistence with people, life activity, and participation in society.	No	(1) ASD symptoms ↓ via dance (P=.01), via equine-assisted therapy (P=.03). and via dance and equine-assisted therapy (P=.02); (2) Functioning independence ↑ via dance (P=.03) followed by communication (P=.01) and psychosocial adjustment (P=.02); (3) Social participation ↑ via dance (P=.04), equine-assisted therapy (P=0.03) and dance associated with equine-assisted therapy (P<.001).

© 2023 Fan MSN et al. *JAMA Network Open.*

Ward et al. (2013)	(1) The Gilliam autism rating scale-2 (GARS-2); (2) The sensory profile school companion (SPSC)<br><br>Rated by teachers (1-2)	(1) The autism index, stereotyped behaviors, communication, and social interaction; (2) SPSC Quadrant (responsiveness to sensory experience, namely registration, seeking, sensitivity, and avoiding); SPSC school Factors (responsiveness manifested in classroom participation, namely School faction1/2/3/4); Section score (Auditory. Visual, movement, touch, and behavior)	No	(1) Effect for time [F (20, 190) =1.97, p<.05]; Autism index ↓ [F (5, 60) =2.43, p<.05]; Social interaction ↓ [F(5, 60)=4.61, p<.05]; stereotyped behaviors→; communication→<br>(2) Effect for time for the quadrant score [F (20, 322.6) =2.03, p<.05]; Registration ↑ [F (5, 100) =2.29, p<.05]; Sensitivity ↑ [F (5, 100) =2.99, p<.05]; Effect for time for the School Factor score [F (20, 322.6) =1.89, p<.05]; School Factor 1 ↑[F (5, 100) =3.42, p<.05]; School Factor 4 ↑[F (5, 100) =2.36, p<.05]; Effect for time for the section score [F (25, 358.1) =2.05, p<.05]; auditory ↑[F (5, 100) =3.42, p=.007]; Visual ↑[F (5, 100) =3.50, p<.05]; Touch ↑[F (5, 100) =3.82, p<.05]
Zachor et al. (2016)	(1) The Social Responsiveness Scale (SRS); (2) The Vineland Adaptive Behavior Scales (VABS); (3) The Teachers' Perceived Future Capabilities Questionnaire.<br>Rated by kindergarten teachers	(1) social awareness, social cognition, social communication, social motivation, and autistic mannerisms; (2) communication, daily living skills, socialization, and motor skills.<br>autism severity; (3) Student's future socio-communication and learning skills.	No	Autism severity →<br>Social awareness→; social cognition ↑, social communication ↑ (p=0.05, η²=0.126), social motivation ↑, and autistic mannerisms ↑<br>Communication↑; daily living skills ↑
Zhao et al. (2021)	(1) The Social Skills Improvement System Rating Scales (SSIS-RS); (2) The Assessment of Basic Language and Learning Skills-revised (ABLLS-R)<br><br>Rated by teacher (1)<br>Rated by parent (2)	(1) communication, cooperation, assertion, responsibility, empathy, engagement, and self-control;	No	(1) Social skills ↑ (Time effect): F=38.874, p<.05, ES=0.573; (Time x group effect): F=21.057, P<.05), ES=0.421; communication ↑ [F(2,58)=10.764, P<.001, ES=0.271], cooperation →, assertion→, responsibility ↑ [F(2,58)=4.168, P=.020, ES=0.126, empathy ↑ [F(2,58)=3.399, P=.04, ES=0.105], engagement→, self-control ↑ [F(2,58)=8.928, P<.001, ES=0.235]; (2) ABLLS-R (Time effect): F=62.915, P<.001, ES=0.684; (Time x group effect): F=31.076, P<.001, ES=.517

[1]→ - No significant change; [2]↑ - significant improvement; [3] ↓ -significant decrease; [4] ❡ - no statistical analysis was conducted

© 2023 Fan MSN et al. *JAMA Network Open.*
