# Supplementary material for: Nature-Based Interventions for Autistic Children: A Systematic Review and Meta-Analysis
Source: JAMA Netw Open. 2023 Dec 7;6(12):e2346715. doi: 10.1001/jamanetworkopen.2023.46715 (PMC10704280; doi:10.1001/jamanetworkopen.2023.46715)
Supplement: Supplement 2. — Data Sharing Statement [file jamanetwopen-e2346715-s002.pdf]

Data Sharing Statement

Fan. Nature-Based Interventions for Autistic Children. *JAMA Netw Open*. Published December 07, 2023. doi:10.1001/jamanetworkopen.2023.46715

Data
Data available: No
